# Connectivity and dynamics in the olfactory bulb

**DOI:** 10.1371/journal.pcbi.1009856

**Published:** 2022-02-07

**Authors:** David E. Chen Kersen, Gaia Tavoni, Vijay Balasubramanian

**Affiliations:** 1 Computational Neuroscience Initiative, University of Pennsylvania, Philadelphia, Pennsylvania, United States of America; 2 Department of Bioengineering, University of Pennsylvania, Philadelphia, Pennsylvania, United States of America; 3 Perelman School of Medicine, University of Pennsylvania, Philadelphia, Pennsylvania, United States of America; 4 Department of Physics and Astronomy, University of Pennsylvania, Philadelphia, Pennsylvania, United States of America; 5 Department of Neuroscience, Washington University in St. Louis, St. Louis, Missouri, United States of America; 6 Department of Neuroscience, University of Pennsylvania, Philadelphia, Pennsylvania, United States of America; Research Center Jülich, GERMANY

## Abstract

Dendrodendritic interactions between excitatory mitral cells and inhibitory granule cells in the olfactory bulb create a dense interaction network, reorganizing sensory representations of odors and, consequently, perception. Large-scale computational models are needed for revealing how the collective behavior of this network emerges from its global architecture. We propose an approach where we summarize anatomical information through dendritic geometry and density distributions which we use to calculate the connection probability between mitral and granule cells, while capturing activity patterns of each cell type in the neural dynamical systems theory of Izhikevich. In this way, we generate an efficient, anatomically and physiologically realistic large-scale model of the olfactory bulb network. Our model reproduces known connectivity between sister vs. non-sister mitral cells; measured patterns of lateral inhibition; and theta, beta, and gamma oscillations. The model in turn predicts testable relationships between network structure and several functional properties, including lateral inhibition, odor pattern decorrelation, and LFP oscillation frequency. We use the model to explore the influence of cortex on the olfactory bulb, demonstrating possible mechanisms by which cortical feedback to mitral cells or granule cells can influence bulbar activity, as well as how neurogenesis can improve bulbar decorrelation without requiring cell death. Our methodology provides a tractable tool for other researchers.

## Author summary

The function of the olfactory bulb (OB) critically depends on connectivity patterns between its excitatory and inhibitory cells. Here, we develop an anatomically grounded algorithm for efficiently determining the connection probability between mitral cells and granule cells in the OB. We use this algorithm to generate a large-scale network model of the OB with characteristic connectivity distributions between cell types, as well as between sister mitral cells. We simulate the network using the dynamical systems approach of Izhikevich for describing neurons, and show how network structure affects GC-mediated processes, including LFP oscillation frequency, lateral inhibition, odor decorrelation, and cortical feedback. Our results suggest how alterations to the OB network through processes like neurogenesis, or via injury or disease, can have significant effects on function.

## Introduction

The olfactory bulb (OB), an important waystation along the olfactory pathway, synthesizes odor input with feedback from higher cortical structures via its complex internal circuitry. This synthesis occurs through interactions between two principal components of the bulb, excitatory mitral cells (MCs) and inhibitory granule cells (GCs), which create a network that reshapes odor information as it passes to cortex. Computational studies are necessary for understanding how this odor information is reshaped, since we lack experimental methods for interrogating this network’s structure-function dependency. However, the sheer number of neurons, encompassing tens of thousands of excitatory cells and millions of inhibitory cells [1]; intricate network architecture [2–4]; and complex spiking dynamics [5–9] make detailed biophysical simulation impractical at large scale. Thus, many studies use random connections or simple distance-dependent functions to establish MC-GC connectivity [10–22] and often study smaller networks on the order of hundreds or even tens of neurons [10, 11, 13, 14, 19, 22–24], allowing for highly complex, conductance-based neuronal frameworks [7]. Other approaches use rate-based or population equations [12, 21, 25] thereby facilitating models with larger numbers of neurons. Together, these studies have shed light on important OB phenomena, such as beta and gamma oscillations [11, 13–15, 17, 20] and effects associated with olfactory discrimination and perceptual learning [12, 19, 21, 26–28].

However, OB function depends heavily on connectivity: particular arrangements of GCs around MCs can dramatically affect its output [8, 29–38]. Likewise, the characteristic spiking dynamics of GCs and MCs can change overall network behavior, e.g., affecting the nature of oscillations that may play an important role in olfactory coding and perception [14, 39]. With this in mind, we have leveraged diverse anatomical and physiological data to build on earlier models and craft an algorithm for generating large-scale, realistic networks of MCs and GC that facilitate studies of emergent OB network behavior.

In short, we inferred the average distribution of dendrites for each cell type from data [8, 40, 41] and modeled the results geometrically. By calculating the intersection between the dendritic distributions of a given MC and GC, we could extrapolate the average number of synapses for the cell pair and in turn calculate a probability of connection. After constructing spatial distributions of MCs and GCs in the OB, we sampled this connection probability for each MC-GC pair to build a large-scale network constrained by the anatomy and featuring a realistic ratio of GCs to MCs [1]. We modeled each cell using the dynamical systems theory developed by Izhikevich, thus reproducing realistic cellular spiking patterns [8, 9, 42, 43]. The resulting network was tractable: a network with nearly 20,000 units could be simulated for tens of thousands of steps (several seconds of real time) in a few hours on a conventional laptop; parallelizing on a server will divide simulation time by roughly the number of processors used.

Our model reproduced important empirical features of the OB, including differential connectivity patterns among sister and non-sister MCs, decorrelation over short timescales, as well as theta, beta and gamma oscillations in the local field potential (LFP). The model makes the surprising, and testable, prediction that cortical feedback inhibition of MCs via GCs is a network property largely independent of which GCs are targeted, an observation with consequences for our understanding of how context modulates odor representations [28, 44–53], and for theories of the functional purpose of granule cell neurogenesis [12, 21, 26, 36]. The model also predicts that beta and gamma oscillations, which are implicated in numerous theories of odor coding and decoding [54–57], are network properties intrinsic to the bulb that can be modified, suppressed, or enhanced by the extent of granule cell activity [14, 23, 39]. Finally, our model offers evidence of how neurogenesis, which is indispensable to olfactory learning and memory formation [35, 58–61], can intrinsically improve bulbar function without invoking the classical model of activity-based apoptotic selection, whose validity has been called into question by recent experiments [62].

## Results

### Deriving the connection probability

**Fig 1:**
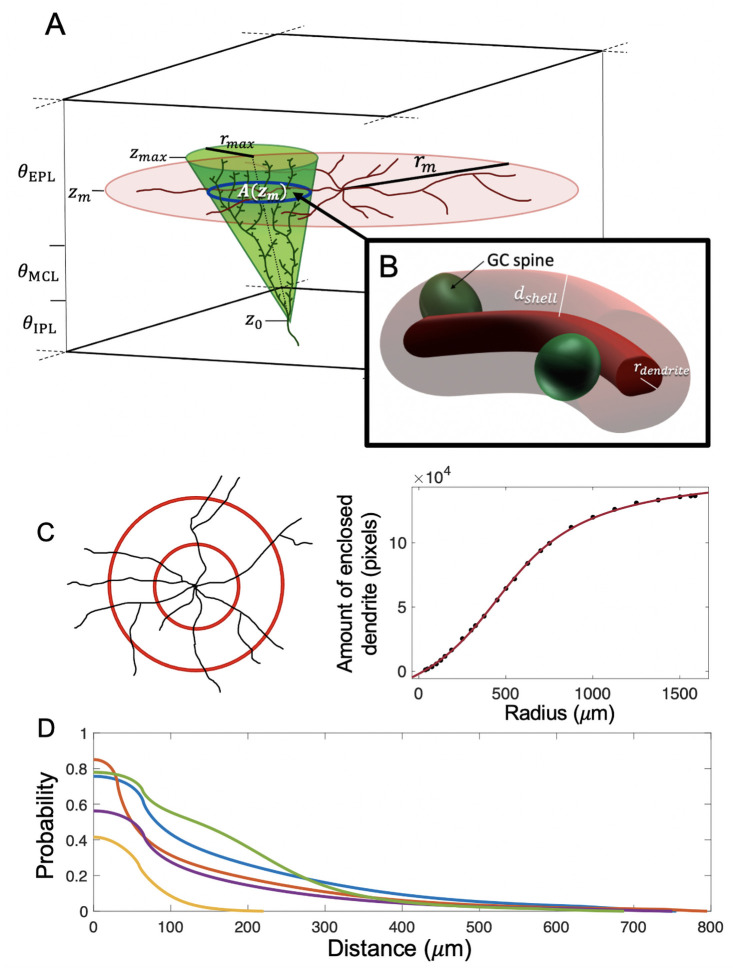
Schematic of the model. (**A**) Our model olfactory bulb (OB) has three layers: the external plexiform layer (EPL), mitral cell layer (MCL), and internal plexiform layer (IPL), each with a specified thickness. We modeled mitral cells (MCs) as flat disks with radius *r*_*m*_ (indicated in red), placed at at a height *z*_*m*_ in the EPL. We modeled granule cells (GCs) as inverted oblique circular cones (indicated in green), with bottom vertex at *z*_0_ in the MCL or IPL, and top face at *z*_max_ in the top half of the EPL; the radius of the top face was *r*_max_. By integrating the MC lateral dendrite density *ρ*_*m*_(*r*) over the area of intersection *A*(*z*_*m*_) between the MC disk and GC cone, we calculated the length of MC lateral dendrite contained in the overlap. (**B**) By treating the lateral dendrites as cylinders, we calculated the potential volume of interaction between MC lateral dendrite and GC spines around the length of MC lateral dendrite in the overlap. Presuming that GC spines are roughly evenly distributed at any given height along the cone, such that the spine density *ρ*_*g*_ depends only on *z*, and that this density is roughly constant over the thickness of the MC disk, the expected number of spines in the interaction volume (which we treat as equivalent to the expected number of synapses) was *ρ*_*g*_(*z*) multiplied by the volume. (**C**) We utilized *camera lucida* images of MCs to calculate the total amount of dendrite within circles of increasing radii and found that this quantity was well fit by an equation *α* tan^−1^(*kr* + *β*) + *C* (mock MC shown). The equation for the density of lateral dendrites *ρ*_*m*_(*r*) was derived from this. For example, a cell image from [41] was well fit by the function with *α* = 66, 270, *k* = 0.002609, *β* = −1.159, and *C* = 55, 010 (*r*^2^ value = 0.9998). (**D**) Sample connection probability curves as a function of distance for different MC/GC pairs, where the connection probability is the probability of at least one synapse existing between a MC-GC pair. This is determined by using the expected number of synapses between that pair as the mean of a Poisson distribution.

To establish the probability that a particular mitral cell (MC) and granule cell (GC) form a connection, we first determined the average number of synapses between that pair. Unlike most neurons, MCs and GCs of the olfactory bulb (OB) form synapses between dendrites, specifically between MC lateral dendrites, which extend out from the soma along the contour of the bulb, and GC dendritic spines, small ellipsoid protuberances off the GC dendrite [63, 64]. The dendritic trees of these cells have stereotypical shapes, so we approximated the OB as a flat 3-dimensional space, and used simple geometric forms to represent the mean spatial distributions of these trees (Fig 1A). The lateral dendrites of MCs extend radially, roughly in a disk when viewed from above [9, 40, 41], so we defined the MC dendritic tree as a radially-symmetric distribution on a flat disk, with each disk oriented parallel to the faces of the OB space. *Camera lucida* images from [40] and [41], showed that the density (in *μ*m of lateral dendrite per *μ*m^2^ area) at a given radial distance *r* from the soma was well fit by the function (Fig 1C):

$$\begin{array}{c} \rho_{m}\left(r\right)=\frac{\alpha k/2\pi r}{1+{\left(kr-\text{tan}\left(m\right)\right)}^{2}} \end{array}$$ (1)

where *α*, *k*, and *m* are constants, and *ρ*_*m*_ lies between 0 and some maximum radius *r*_max_ (derivation in Methods).

GC dendrites pass roughly orthogonally to MC lateral dendrites, with the effective radius of the dendritic tree increasing along the height of the tree [8, 9, 40, 41]. We therefore defined the GC dendritic tree over an inverted oblique circular cone, with face parallel to the faces of the OB space. The volumetric spine density (number of spines per *μ*m^3^) at a given height *z* on the cone is calculated from dividing the vertical spine density *N*_*s*_ (defined as the number of spines per *μ*m height) by the cross-sectional area of the cone at *z* (Eq 32):

$$\begin{array}{c} \rho_{g}\left(z\right)=\frac{N_{s}\left(z\right)}{\pi r{\left(z\right)}^{2}} \end{array}$$ (2)

where *ρ*_*g*_ is defined over a range between some minimum and maximum heights *z*_0_ and *z*_max_ marking the bottom and top of the cone respectively. Since the vertical spine density of GCs tends to have an overall concave shape [8, 40], we modeled our vertical spine density as a simple parabola following [8, 40]. *ρ*_*g*_(*z*) can then be expressed as:

$$\begin{array}{c} \rho_{g}\left(z\right)=\frac{-6S}{\pi r_{\text{max}}^{2}\left(z_{\text{max}}-z_{0}\right)}(\frac{z-z_{\text{max}}}{z-z_{0}}) \end{array}$$ (3)

where *r*_max_ is the maximum cone radius and *S* is the total number of available spines on the GC (derivation in Methods).

We defined the average number of synapses between a MC-GC pair as the average number of GC spines within sufficient proximity to the lateral dendrites of an MC to establish synapses. Using the above distributions, we first calculated the length *L* of MC lateral dendrites present in the overlap between the MC and GC dendritic trees by integrating the MC dendritic density over the area of intersection between the two trees (Eq 38):

$$\begin{array}{c} L=\int_{A(z_{m})}\rho_{m}\left(r\right)dA=\underset{A(z_{m})}{\;\int \int \;}\frac{\alpha k/2\pi r}{1+{\left(kr-\text{tan}\left(m\right)\right)}^{2}}rdrd\theta \end{array}$$ (4)

Here *A*(*z*_*m*_) is the area of intersection between the MC disk and the GC cone at the disk’s height *z*_*m*_. The integrals for the different cases, which depend on the relative position and sizes of the MC and GC, are shown in Methods.

In order to account for the 3-dimensional nature of potential interactions between MC and GC, we converted the overlap dendritic length *L* into an equivalent volume by assuming the lateral dendrites to be cylinders of radius around 0.63 *μ*m [9]. We then defined the volume of interest to be the cylindrical sheath of thickness *d*_shell_ surrounding the lateral dendrite, with *d*_shell_ = 1.02 *μ*m, the effective diameter of a spine [64] (Fig 1B). The volume of this sheath was then:

$$\begin{array}{c} V=\pi ({\left(d_{\text{shell}}+r_{\text{dendrite}}\right)}^{2}-r_{\text{dendrite}}^{2})L=q\pi L \end{array}$$ (5)

with *q* = 2.32 *μ*m^2^.

The density of GC spines in this volume was *ρ*_*g*_(*z*_*m*_), under the simplifying assumption that it was constant throughout the sheath volume. Thus, the expected number of spines in this volume, and by our definition synapses, was just:

$$\begin{array}{c} \lambda =\rho_{g}\left(z_{m}\right)V=q\pi \rho_{g}\left(z_{m}\right)L \end{array}$$ (6)

We used a Poisson distribution to calculate the connection probability from the expected number of synapses. Because most MC-GC pairs make only one synapse [64] (and indeed, from our algorithm, the expected number of synapses between a given MC and GC was almost always less than one), we limited MCs and GCs to forming at most 1 connection. To that end, we treated the Poisson probabilities of multiple synapses forming as contributing to the overall probability of a single connection forming. In short, our connection probability was the probability that at least 1 synapse existed between a MC-GC pair:

$$\begin{array}{c} P\left(\text{connection}\right)=P\left(N_{\text{synapse}}\neq 0\right)=1-\text{exp}\left(-\lambda \right) \end{array}$$ (7)

with a connection being treated as a single synapse.

### Cell placement

To spatially distribute MCs and GCs, we modeled the OB as a thin circular cylinder with area *A* and thickness *θ* subdivided into parallel layers based on OB anatomy [65]. MC disks were distributed in the topmost layer of our model, the equivalent of the external plexiform layer (EPL), where interactions between GCs and MCs occur [2]. The number and location of these MCs was determined by their glomeruli, which are conglomerations of the apical dendrites of MCs. These glomeruli existed in the eponymous glomerular layer (not modeled) above the EPL, and we assumed that projections of these glomeruli onto the EPL were distributed randomly in the x-y plane, an easily relaxed assumption if future connectomic studies provide finer information. We calculated from data the overall area density of glomeruli in the OB (Methods), so the number of glomeruli for a given OB space was just this density multiplied by the area *A*. Each glomerulus was randomly assigned between 15 to 25 MCs, whose disk centers were randomly scattered in the x-y plane around that glomerulus’s projection, with distance from the glomerular projection drawn from a truncated logistic distribution fit to data from [4] (S1 Fig). MCs were also divided into type I and type II subgroups (2:1 ratio), which differ in where in the EPL their lateral dendrites ramify [2, 40, 41]; thus the z-position of each MC in our model depended on its type assignation. The radius of an MC disk was drawn from a uniform distribution between 75 and 800 *μ*m, based on measurements of MC images from [9].

Once we placed the MCs, we added GCs. GCs can be divided into two major types based on where in the EPL their trees principally spread: the aptly named superficial and deep GCs, which are thought to interact primarily with tufted cels (TCs) and MCs, respectively [8, 66]. Since our excitatory population consisted of MCs, we only included deep GCs, although superficial GCs and TCs could easily be modeled too (see Discussion).

The vertices of the previously described GC cones were distributed uniformly randomly in the x-y plane of the OB space. We used images of GCs from [8] to roughly determine the bounds of GCs in the z-direction. The bottom vertex of the cone either inhabited the mitral cell layer (MCL), which lies directly below the EPL, or the internal plexiform layer (IPL), which is below the MCL and constituted the bottom-most layer of our model. Meanwhile, the top face of the cone was confined to the top half of the EPL, and the xy-position of its center was located a random distance away from the xy-position of the bottom vertex (*i.e.* potentially making the cone oblique).

For simplicity, we drew the total number of spines *S* for a particular GC from a uniform distribution determined by the cone’s volume. However, because only spines in the EPL are relevant for forming synapses with MCs, we limited the number of possible synapses each GC could make (*S*_available_) to the total number of spines present in the EPL, which could be found by integrating *N*_*s*_(*z*) from the bottom of the EPL to the maximum height of the cone:

$$\begin{array}{c} S_{\text{available}}=\int_{\theta_{\text{IPL}}+\theta_{\text{MCL}}}^{z_{\text{max}}}N_{s}\left(z\right)dz \end{array}$$ (8)

### Network generation

To generate the network, we added GCs individually and compared with each MC to determine, via the equations above, whether a connection would exist between that pair. To account for preexisting synapses that an MC might already have (since spines corresponding to those synapses occupy space in the interaction volume surrounding the MC lateral dendrites), we weighted the calculated volume *V* = *qπL* for that MC-GC pair by the ratio of the volume of unoccupied interaction space to the volume of total interaction space on that MC, assuming for simplicity that the preexisting synapses are distributed evenly along the length of the dendrites:

$$\begin{array}{c} V_{\text{eff}}=V(1-\frac{N_{\text{ps}}V_{\text{spine}}}{V_{\text{tot}}}) \end{array}$$ (9)

Here *N*_ps_ is the number of preexisting synapses, *V*_tot_ is the total volume of interaction space on the MC, and *V*_spine_ = 0.58 *μ*m^3^ is the average volume of a spine [64]. We used *V*_eff_ in Eq 6 to determine the average number of spines and in turn the connection probability. We repeated this for each MC (whose order is shuffled for each new GC to avoid bias) until every MC in range has been tested. If the number of connections exceeded the maximum number of synapses allowed for that GC, we retained a random subset of those connections with size equal to the number of available synapses, and removed the remnant. Since the network generation was probabilistic, it was unlikely but not impossible that a GC would be disconnected from all mitral cells and thereby not contribute to the network. Thus, we generated GCs individually, discarding those that were disconnected from all mitral cells, until we reached a target number of GCs, which we calculate to be 15 deep GCs per MC based on current estimates of cell numbers in the OB [1]. Thus, we generated a model OB of radius 600 *μ*m (area 1.13 mm^2^) containing a network of 3,550 MCs and 53,250 GCs.

### Single cell dynamics

To explore network function we modeled individual MCs and GCs as dynamical systems described by the Izhikevich equations [42, 43]:

$$\begin{array}{c} C\frac{dv}{dt}=k\left(v-v_{r}\right)\left(v-v_{t}\right)-u+I \end{array}$$ (10)

$$\begin{array}{c} \frac{du}{dt}=a\left(b\left(v-v_{r}\right)-u\right) \end{array}$$ (11)

with spike reset:

$$\begin{array}{c} \text{If}\;v\geq v_{c},\text{then}\;\{\begin{array}{c} v\leftarrow c\\ u\leftarrow u+d \end{array} \end{array}$$

where *v* is the membrane potential; *u* is a recovery current; *v*_*r*_ is the resting potential; *v*_*t*_ is a threshold; *v*_*c*_ is a cutoff; *I* is an external current; and *a*, *b*, *c*, *d*, and *k* are free parameters.

**Fig 2:**
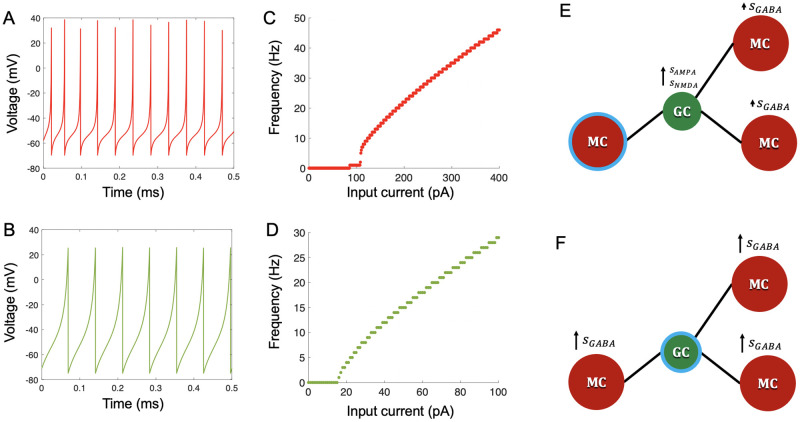
Cell dynamics modeling. (**A**) Sample voltage trace of an MC. Input was 200 pA direct current. (**B**) Sample voltage trace of a GC. Input was 45 pA direct current (**C**) Frequency-current (f-I) curve for an MC. (**D**) f-I curve for a GC. Each cell in (**C**) and (**D**) was simulated with the given parameters in Table 1, receiving direct current for 1 second of in-simulation time per current intensity. (**E**) When an MC spikes (MC with blue ring), gating variables of GC AMPA and NMDA receptors increase at synapses between that MC and all connected GCs. In turn, gating variables of MC GABA receptors increase by a smaller amount at synapses between connected GCs and their subsequent connected MCs (**F**) When a GC spikes (GC with blue ring), gating variables of MC GABA receptors increase at synapses between that GC and all connected MCs.

We selected parameters to model class II behavior of MCs [67] and to establish realistic *f* − *I* curves [9] (Fig 2C). Following conductance based models [5, 7], we took GCs to be integrators [42, 43]. Other work suggests that some GCs may display resonator properties, including subthreshold membrane potential oscillations [68, 69], but the very low oscillation frequency may make them irrelevant to excitability classification [43], especially since some GCs appear to be entirely non-resonant [68]. So, *b* < *a* in the Izhikevich model; additionally, by assuming *b* to be negative, we could take advantage of the following equations to calculate *b* and *k* [43]:

$$\begin{array}{c} b=\frac{v_{r}-v_{t}+4R\rho }{4R^{2}\rho } \end{array}$$ (12)

$$\begin{array}{c} k=\frac{1}{4R^{2}\rho } \end{array}$$ (13)

where *R* is input resistance and *ρ* is the rheobase (minimum DC current to produce spikes). We chose the remaining parameters to match a realistic *f* − *I* curve from [8] (Fig 2D).

The parameter values are given in Table 1. We drew parameters from a normal distribution with standard deviations equal to 1/10 of the absolute value of each mean, except for *b* and *k* for GCs. We drew the latter two parameters from normal distributions with standard deviations equal to 2/3 of the absolute value of each mean under the constraint that *b* < 0, and with each pair of these parameters for each cell being re-sampled until the rheobase for each GC was between 10 and 70 pA, and the input resistance for each GC was between 0.25 and 1.5 G*Ω*.

**Table 1:** Parameters for single cell internal dynamics.

Variable	Mitral cell	Granule cell
*k* (nS(mV)^−1^)	2.5	0.067
*a* (ms^−1^)	0.02	0.01
*b* (nS)	12	-0.133
*c* (mV)	-70	-75
*d* (pA)	13	2
*v*_*r*_ (mV)	-58	-71
*v*_*t*_ (mV)	-49	-39
*v*_*c*_ (mV)	30	25
*C* (pF)	191	48

### Synaptic dynamics

We modeled dendrodendritic synapses for MC-GC pairs as NMDA and AMPA receptors on GCs, and GABA receptors on MCs. The synaptic AMPA current was

$$\begin{array}{c} I_{\text{AMPA}}\left(t\right)=s\left(t\right)\;g_{\text{AMPA}}\;\left(V\left(t\right)-E_{e}\right) \end{array}$$ (14)

where *g*_AMPA_ is the conductance, *s*(*t*) is a gating variable representing the fraction of open channels, *V*(*t*) is the voltage of the recipient cell, and *E*_*e*_ = 0 mV is the excitatory reversal potential. For GABA receptors, we also noted that inhibitory signals from the cell periphery degrade as they propagate to the soma [70], which we described as an exponential decay. Including this decay, we modeled the GABA current as

$$\begin{array}{c} I_{\text{GABA}}\left(t\right)=s\left(t\right)\;g_{\text{GABA}}\;\left(V\left(t\right)-E_{i}\right)\text{exp}\left(-\frac{L}{\lambda }\right)\;, \end{array}$$ (15)

where *E*_*i*_ = −70 mV is the inhibitory reversal potential, *L* is the distance between the MC center and the synapse, λ is a length constant, and other parameters were as for AMPA. The network generation did not identify MC-GC synapsse locations, so we chose points sampled randomly from the overlap between each MC and GC.

For NMDA receptors, we used [71]:

$$\begin{array}{c} I_{\text{NMDA}}\left(t\right)=s\left(t\right)\frac{g_{\text{NMDA}}\;\left(V\left(t\right)-E_{e}\right)}{1+\frac{[{\text{Mg}}^{2+}]\text{exp}\left(-0.062V(t)\right)}{3.57}} \end{array}$$ (16)

where the additional term in the denominator describes the magnesium block, with [Mg^2+^] assumed to be 1 mM [72].

The AMPA and GABA gating variables evolved as [73]:

$$\begin{array}{c} \frac{ds_{X}}{dt}=\frac{-s_{X}}{\tau_{X}}\;, \end{array}$$ (17)

where *X* = GABA or AMPA. The NMDA dynamics followed

$$\begin{array}{ccc} \frac{ds_{\text{NMDA}}}{dt} & = & \frac{-s_{\text{NMDA}}}{\tau_{{\text{NMDA}}_{\text{decay}}}}+\alpha n\left(1-s_{\text{NMDA}}\right)\;,\\ \frac{dn}{dt} & = & \frac{-n}{\tau_{{\text{NMDA}}_{\text{rise}}}}\;. \end{array}$$ (18)

MC activation at reciprocal MC-GC synapses causes excitatory glutamate release onto NMDA and AMPA receptors on GCs, while GC activation causes inhibitory GABA release onto GABA receptors on MCs [74, 75]. Thus, when an MC spiked, the gating variables of GC NMDA and AMPA receptors at its synapses were updated as (Fig 2E, center GC):

$$\begin{array}{ccc} s_{\text{AMPA}} & \leftarrow & s_{\text{AMPA}}+W\left(1-s_{\text{AMPA}}\right)\\ n & \leftarrow & n+W(1-n)\;, \end{array}$$ (19)

where *W* = 0.5. To account for network-driven GC activity [6], GABA gating variables of MCs indirectly connected to a spiking MC via shared GCs were also updated: (Fig 2E, right):

$$\begin{array}{c} s_{\text{GABA}}\leftarrow s_{\text{GABA}}+\kappa W\left(1-s_{\text{GABA}}\right) \end{array}$$

where 0 < *κ* < 1. When a GC spiked, the gating variables of MC GABA receptors at its synapses were updated as (Fig 2F):

$$\begin{array}{c} s_{\text{GABA}}\leftarrow s_{\text{GABA}}+W\left(1-s_{\text{GABA}}\right) \end{array}$$

For each cell, the synaptic input at any time was the sum of the currents for its receptors at all synapses (NMDA and AMPA for GCs, GABA for MCs). We derived time constants *τ* and *α* from data [74], and tuned conductances and *κ* to reproduce lateral inhibition results from [76] as faithfully as possible. We calculated the length constant λ from the formula in [70] for the diameter of dendrite used in the connectivity algorithm (*d* = 1.26 *μ*m). Parameters are in Table 2.

**Table 2:** Synaptic parameters.

Parameter	Value
*g*_AMPA_ (nS)	0.73
*g*_NMDA_ (nS)	0.84
*g*_GABA_ (nS)	0.13
*κ*	0.006
*τ*_AMPA_ (ms)	5.5
$\tau_{{\text{NMDA}}_{\text{rise}}}(\text{ms})$	10
$\tau_{{\text{NMDA}}_{\text{decay}}}(\text{ms})$	80
*τ*_GABA_ (ms)	18
λ (*μ*m)	675
*α* (ms^−1^)	0.1

### Summary of the algorithm

Altogether, to generate and simulate a network, we can distill our method into the following steps:

1. We select a radius of the OB space. This will determine the number of glomeruli, MCs, and GCs in the system. Correspondingly, the glomerular density, the number of MCs per glomerulus, and the number of GCs per MC can all be adjusted as desired.
2. The projections of the glomeruli onto the OB space are randomly distributed in the x-y plane. An appropriate number of MCs is assigned to each glomerulus, and their x-y positions are distributed about the center of each glomerular projection, with the range of z-positions being dependent on the MC type. GCs are randomly placed in both the x-y plane as well as along the z-axis.
3. Each MC or GC is assigned parameters detailing the mean spread of its dendrites.
4. Each MC-GC pair which physically overlap is evaluated to determine the connection probability between the two. This probability is then treated as a Bernoulli variable and sampled accordingly to determine if a connection exists between the pair. Because of the limited number of spines possessed by each GC which can be used to form synapses, if a GC makes more synapses than is permissible in this way, a random subset of its connections is removed.
5. Ultimately, from these connections we extrapolate a binary matrix containing the connectivity data of the network.
6. To simulate the network, each MC and GC is assigned parameters for the Izhikevich model. Although we use this specific model, other neuronal models, such as the leaky integrate-and-fire, could be substituted with their own appropriate parameters. Additionally, synaptic parameters are selected for each possible MC-GC synapse.
7. With the network connectivity established and the dynamic parameters selected, we perform experiments.

### Sister mitral cells are weakly correlated in the network

**Fig 3:**
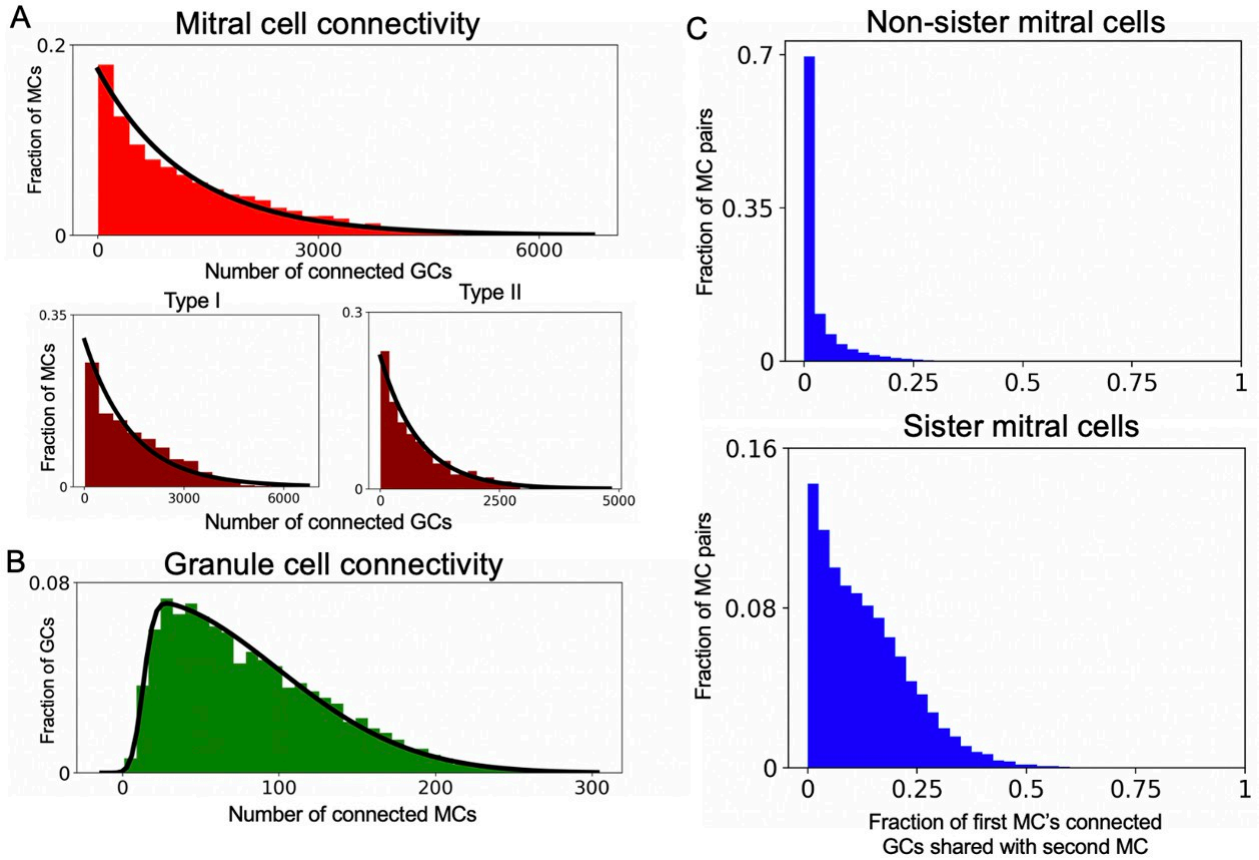
Network connectivity. (A) Top: Distribution of MCs by number of connected GCs, black line = exponential fit (mean 1225.8). Bottom: Distribution of type I (left) and II (right) MCs by number of connected GCs. Black line for type I MCs = exponential fit (mean 1426.0). Black line for type II MCs = exponential fit (mean 818.7). (B) Distribution of GCs by number of connected MCs. Black line = skewed normal fit (parameters *α* = 15.2, *ξ* = 13.0, *ω* = 85.5; see Methods for definition of skewed normal) (C) Distribution of fraction of connected GCs shared with another MC for non-sister (top) and sister (bottom) MCs.

We asked what our local connection rules predicted for global network features such as MC to GC connectivity and vice versa. The distribution of MC connectivity to GCs was well fit by an exponential (Fig 3A, top), as were individual type I and type II distributions (Fig 3A, bottom). However, type I MCs connected to more GCs than their type II counterparts, likely because type II MC dendrites ramify higher in the EPL and thus overlap less with deep GC dendritic baskets. The distribution of GC connectivity to MCs was well fit by a skewed normal distribution (Fig 3B; see Methods). These structural predictions can be tested as bulb connectomes become available. We also found that sister MCs, i.e., MCs connected to the same glomerulus, connected to more of the same GCs than non-sister MCs, but, surprisingly, this overlap was low (mean = 0.13) (Fig 3C). This predicts that sister MCs, despite originating in the same glomerulus, will have distinct lateral dendrite synaptic patterns consistent with [4], and will encode odors non-redundantly, perhaps explaining findings in [3, 77].

### Network oscillations and granule cell inhibition

There are prominent local field potential (LFP) oscillations in the OB, with different frequencies associated to specific aspects of olfaction, e.g., fine odor discrimination and associative learning [55]. Thus, we tested whether odor input produced oscillations, and in what range. MCs received Poisson inputs with time-varying rates [78] from olfactory sensory neurons (OSNs) activating 100 synapses per MC, each with an NMDA and AMPA receptor modeled as Eqs (17) and (18), albeit with conductances and time constants based on [79] (Table 3). For each synapse, a spike input caused the NMDA and AMPA gating variables to increase as in (19).

**Table 3:** External input parameters.

Parameter	Value
*g*_AMPA_ (nS)	6.7
*g*_NMDA_ (nS)	12
*τ*_AMPA_ (ms)	14.3
$\tau_{{\text{NMDA}}_{\text{rise}}}(\text{ms})$	13
$\tau_{{\text{NMDA}}_{\text{decay}}}(\text{ms})$	70
*α* (ms^−1^)	0.03

To simulate odor input, we modeled the Poisson rate *r*(*t*) for the external inputs as

$$\begin{array}{c} r\left(t\right)=\frac{r_{\text{max}}}{2}+\frac{r_{\text{max}}}{4}\left(\text{sin}\;\left(2\pi ft-\phi \right)+1\right)\;. \end{array}$$ (20)

with *f* = 6 Hz, representing the sniff rate. We selected 20% of the glomeruli to receive odor input. To determine *r*_*max*_ for these odor-receiving glomeruli, we drew an initial mean glomerular rate *x*_*g*_ uniformly from 2 to 3 Hz for each glomerulus; then for each sister MC of this glomerulus, we drew *r*_*max*_ from a Gaussian with mean *x*_*g*_ and standard deviation *x*_*g*_/10. Similarly, to determine the phase *ϕ*, we first drew an initial mean glomerular phase *p*_*g*_ uniformly from 0 to 2*π* for each glomerulus, since phases of non-sister MCs are highly uncorrelated [77]. Then for each MC in a glomerulus we drew *ϕ* from a Gaussian with mean *p*_*g*_ and standard deviation *π*/4, reflecting variability among sister MCs [77]. The process was repeated in the same fashion for non-odor-receiving glomeruli, except the range *x*_*g*_ was lowered to be 0 to 0.25 Hz.

**Fig 4:**
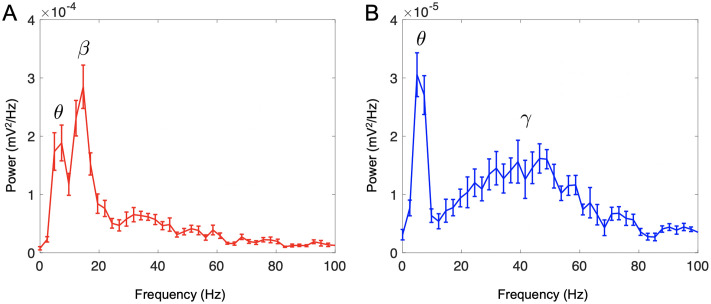
Oscillatory dynamics. (A) A network with a 15:1 GC:MC ratio. Odor input to a subset of glomeruli caused oscillations at ∼15 Hz, in the lower beta range (15–40 Hz), in addition to activity at 6 Hz, corresponding to respiration in the theta range (2–12 Hz). (Bottom). (B) A network simulating tonically inhibited GCs, and hence a lower 5:1 active GC:MC ratio. Odor input caused oscillations with a broad frequency peak around 40–55 Hz, in the gamma range (35–100 Hz) as well as activity at 6 Hz, corresponding to respiration in the theta range. Curves averaged from 10 trials; bars indicate standard error.

We used [80, 81] to calculate LFPs:

$$\begin{array}{c} \phi \left(r_{e},t\right)=\sum_{s=1}^{S}\frac{I_{s,\text{AMPA}}\left(t\right)+I_{s,\text{NMDA}}\left(t\right)+I_{s,\text{GABA}}\left(t\right)}{4\pi \sigma |r_{e}-r_{s}|} \end{array}$$ (21)

where for each synapse *s*, **r**_*s*_ is the location, *I*_*s*,*X*_(*t*) is the current through a receptor type, and *σ* is extracellular conductivity (1/300 *Ω*^−1^ cm^−1^). Here, **r**_*e*_ is the “electrode” location at the xy-center and halfway up the EPL. After filtering and selecting the region of interest, we acquired the LFP power spectrum averaged across 10 trials (Methods; Fig 4).

During odor input, the LFP oscillated at 6 Hz, corresponding to the sniff rate, and at 15 Hz, in the beta range (Fig 4A). This was surprising, since odor presentation is thought to produce gamma oscillations (35–100 Hz) due to activity at the MC-GC synapse, while beta oscillations are more commonly associated with cortical feedback to the OB [14, 55]. However, given that a major role of cortical feedback is to activate GCs [82], and that without such feedback, large segments of GCs are tonically inactivated [1, 38, 83], we hypothesized that reducing the number of active GCs to approximate tonic inhibition might produce gamma oscillations, especially since other computational studies demonstrating gamma have utilized lower GC:MC ratios [11, 14]. Therefore we repeated the experiment with a GC:MC ratio set to $\frac{1}{3}$ that of the full network. Indeed, odor presentation led to LFP oscillations peaking around 40–55 Hz, in the gamma range (Fig 4B). This suggests that overall activity of the GC network, determined by a balance between tonic inhibition and excitatory feedback, is a major determinant of whether the OB oscillates in the beta or gamma ranges during odor presentation [84, 85]. This is in line with studies demonstrating the importance of GC excitability to LFP oscillation frequency [14, 23, 39].

### Lateral inhibition follows network architecture

**Fig 5:**
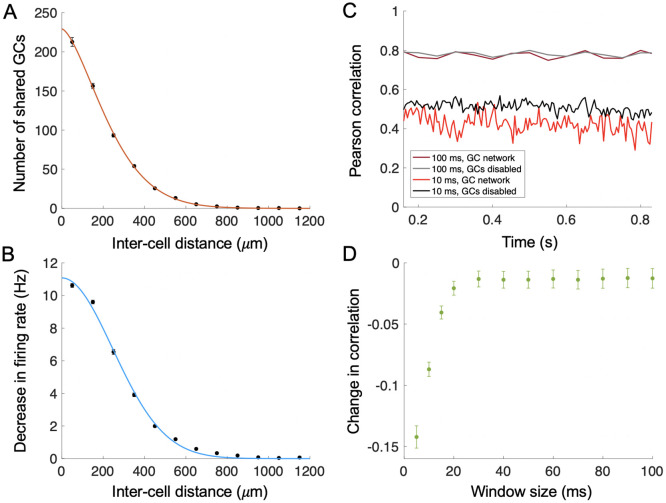
Network connectivity, lateral inhibition, and decorrelation. (A) The number of shared GCs between pairs of MCs (*n* = 1436). MC pairs were partitioned based on inter-cell distance into 12 bins each representing a 100 *μ*m span. Each point represents the average number of shared GCs of all MC pairs in a given bin and was placed halfway along the bin width, with the bar representing standard error. The red curve is a fit of the form *a* exp(−*bx*^*n*^), with *x* in microns, *a* = 229.2, *b* = 1.721e- 04 and *n* = 1.545. (B) Lateral inhibition strength decreases with distance between MCs. MC pairs (*n* = 1436) were simulated with one and then both cells receiving current input, and the resulting decrease in firing rate for the first cell was measured. MC pairs were again partitioned based on inter-cell distance into 12 bins each representing a 100 *μ*m span. Each point represents the average decrease in firing rate of all MC pairs in a given bin and was placed halfway along the bin width, with the bar representing standard error. The blue curve represents a fit of the form *a* exp(−*bx*^*n*^), with *a* = 11.08, *b* = 9.375e- 06 and *n* = 1.976. (C) Example of evolution of Pearson correlation between model OB responses to two odors, each targeting 30 of 178 glomeruli (25 overlapping) over time, in the presence and absence of GC activity. Dark red/grey: GCs enabled/disabled for 100 ms time window. Bright red/black: GCs enabled/disabled for 10 ms time window. (D) The decrease in Pearson correlation was maximal for shorter timescales. We measured mean correlation over the period after the first sniff ($\frac{1}{6}$ of a second) for networks with and without GCs, and then calculated the difference of the two. The magnitude of decorrelation increased as the window size decreased below 20 ms. Data averaged over *n* = 15 odor pairs, each odor activating 30 glomeruli, and with varying overlap ranging from 5 to 25 glomeruli (average overlap = 18). Error bars = standard error of the mean.

It is believed that lateral inhibition by granule cells may be involved in gain control, synchronization of MC output, and decorrelation of odor representations [34, 76, 86–91]. To ask how recurrent interaction between MCs and GCs varied with distance, we first measured the number of GCs shared between pairs of MCs at around the same height in the EPL, and connected individually to similar number of GCs. This shared number decreased with distance following a relation of the form *a* exp(−*bx*^*n*^) (Fig 5A), with *n* ∼ 1.5 intermediate between an exponential and a Gaussian, leading us to anticipate that lateral inhibition between pairs should be of similarly short range. To test, we selected MC pairs as above, and mimicked the experimental methodology of [76], where we measured the firing rate of one MC when (a) it alone was excited with direct current and (b) when the other MC of the pair was also excited, with the firing rates being measured over 1 s time intervals. We found that the decrease in firing rate of the first cell in conditions (a) vs. (b) declined with separation between the pair and had a similar short-range form to the relationship between MC separation and number of shared GCs (Fig 5B). For comparison, we built a second network with the same number of cells and average MC-GC connectivity but with constant, distance-independent probability of connection for each MC-GC pair. In this second network, both the number of shared GCs and magnitude of lateral inhibition were constant and independent of distance (S2 Fig). This suggests that the assumption of random connectivity used for simplicity in many studies [10, 20, 21, 23] leads to fundamentally different inhibitory effects in the feedforward olfactory pathway.

Given that the magnitude of the decrease in firing rate for an MC produced through lateral inhibition was relatively low (Fig 5B) compared to the firing rate produced through direct current stimulation of the MC alone (∼70 Hz), we were curious whether the GC network in our model could effectively decorrelate odor patterns. To answer this question, we generated a set of 6 odors, with odor 1 targeting glomeruli 1 through 30, odor 2 targeting 6 through 35, and so on, leading to an average overlap between odors of ∼18 glomeruli; we then simulated our system receiving each of these odors. We ensured that odor inputs for the overlapping glomeruli were delivered at the same phase and strength, because input phase differences can already decorrelate responses, and we were primarily interested in the specific role played by the GC network. We measured the Pearson correlation of the MC firing rates induced by each odor within sliding time windows of fixed duration. Afterward, we repeated the same experiment, except with the GC network disabled by setting the GABA conductances on MCs to zero (Fig 5C). Then, for each condition we time-averaged the correlation after the first sniff (by which point the system had equilibrated). Finally, we calculated the difference between the odor-response correlations measured with and without the granule cell network, and treated this as the amount of decorrelation induced by the GC network. Without granule cells (black and grey lines in Fig 5C), we found that the MC output correlation reflected the number of overlapping glomeruli when measured over long time windows, but was less correlated over short windows because of variations in spike timing for individual MCs determined by the dynamics. We then assigned a single correlation value to each odor pair by taking the mean of the correlation values for the time windows comprising the second sniff. When comparing these single values for each odor pair with and without the GC network we observed that, at short time scales, this GC network had a small but significant decorrelating effect, especially when the response correlation was measured in windows of <20 ms (Fig 5C and 5D). This is consistent with experiments showing that GCs primarily produce decorrelation by altering spike timing along these short timescales [92].

### Network architecture shapes cortical feedback

GCs and MCs receive extensive cortical feedback, which plays a major role in shaping odor representation as information is conveyed through the OB to cortex [28, 82, 93–97]. We thus asked how the arrangement of GCs in the network determined the expression of this feedback in the OB. We first examined the effect of external activation of the GC network on odor-receiving MC output, and how this effect varied with the spatial pattern of GC activation. Thus, we targeted excitatory feedback randomly to between 0.1%-20% of all GCs, rounded to the nearest integer, during presentation of an odor, and then compared to simulations where no feedback was present. The first two trials used the same network but targeted non-overlapping sets of GCs, in order to ascertain whether the effect of feedback could be attributed to which set of GCs was targeted. In the third trial, we performed the same experiment but in a different network which had the same arrangement of MCs but a different configuration of GCs. We calculated the change in odor-receiving MC firing rates with feedback for each trial and then computed the mean correlation of these changes over time between the first and second trials (same GC network) and between the first and third trials (different GC networks) using a sliding window of length 10 ms and 50% overlap between windows.

**Fig 6:**
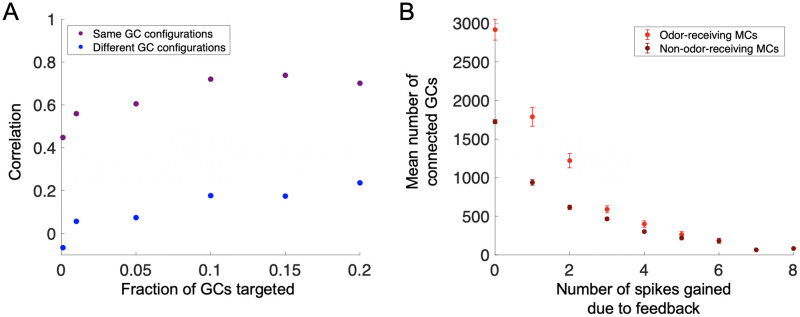
Network architecture and the effects of external feedback to the OB. (A) The effects of randomly distributed, excitatory feedback to GCs depend on the OB network’s inherent connectivity and are relatively invariant to the specific GCs targeted. We presented an odor to the network without and then with external feedback to GCs, and measured the change in MC firing rate. For each feedback level, we ran three trials; the first two took place in the same MC-GC network, but targeted non-overlapping sets of targeted GCs. The third trial took place in a second network with the same MCs but a different spatial configuration of GCs. We computed the Pearson correlation for successive time windows of length 10 ms between the vector of feedback-driven changes in odor-receiving MC firing rates for the first two trials, in the same network (purple), and the first and third trial, in different networks (blue). We found that even for small numbers of targeted GCs, the mean correlation over time was high for the same-network trials despite targeting different sets of GCs, but was lower for the different-network trials. (B) MCs which connect to the fewest GCs are most affected by direct excitatory feedback. We randomly distributed excitatory feedback to 20% of MCs during odor presentation and recorded the resulting change in firing rate. MCs with larger firing rate increases tended to connect to a smaller number of GCs for both odor-receiving (red) and non-odor-receiving (dark red) MCs. Vertical lines indicate standard error. Data compiled from *n* = 5 trials.

To our surprise, the particular arrangement of the feedback in terms of which GCs were selected had little effect on which MCs were ultimately affected, since the average correlation between trials in the same GC network (Fig 6A, purple) was relatively high even for small amounts of targeted GCs. Moreover, the average correlation values between trials in different GC networks was significantly lower in comparison (Fig 6A, blue). Thus, our results suggest that a primary determinant of the effect of cortical feedback on the bulb output is the network architecture of GCs, as opposed to which of these cells are specifically targeted. This implies that theories of feedback and neurogenesis in the bulb that rely on specific targeting of GCs and MCs [12, 28, 98] may require additional components beyond the basic MC-GC network to be feasible. Interestingly, however, when we used this model in a recent study to examine the effect of GC feedback on cortical odor representation, we found that even highly correlated feedback patterns to GCs in a given network configuration produced robust odor discrimination following cortical transformation of the OB output [53]. This suggests that differential inhibition of MC firing may not be necessary to produce meaningful separation of odor patterns in cortex.

We also examined how changes in MC firing rate due to direct positive feedback depended on the arrangement of GCs in the OB [99, 100], by first presenting the OB with an odor, and then presenting the same odor with excitatory feedback current to a randomly selected set of MCs. Repeating this experiment for different odors, we found that cells whose firing rates increased the most with feedback were connected to the least number of GCs, both for odor-receiving (Fig 6B, red) and non-odor-receiving MCs (Fig 6B, dark red). Thus, just as with GC feedback, the effect of direct MC feedback also appears to depend on the particular architecture of the GC network around MCs.

### Neurogenesis improves odor discrimination without invoking an activity-dependent selection model

Adult neurogenesis is a rare phenomenon in the mammalian central nervous system, with the GCs of the OB being one of the few neuron types which undergo this process. Despite this rarity, GC neurogenesis appears to be indispensable to olfactory learning and memory formation [35, 58–61], in particular strongly enhancing the OB’s ability to facilitate odor discrimination [35, 101, 102]. In the conventional account, experimental evidence is believed to suggest that newborn GCs 1) localize randomly in the OB; 2) “compete” for survival during a critical period, with the most active cells, as determined by the current odor environment, surviving; and 3) replace pre-existing GCs, leading to constant GC turnover [29, 31, 36, 59, 103–108]. Together, this understanding of neurogenesis has been classically referred to as “activity-based selection.” However, recent evidence has demonstrated that the use of high, toxic concentrations of radioactive tracers for labeling newborn GCs has led to a gross overestimation of the amount of cell death these cells undergo, to the point that reduction of these concentrations lead to virtually no cell death at all [62]. These new findings, along with our previous results demonstrating the importance of network structure to OB function, led us to employ our model to investigate if neurogenesis, in the absence of cell death, could still translate into improved OB functionality, as measured by its ability to decorrelate odor input.

In terms of our basic experimental framework, we began with a smaller network organized into 44 glomeruli. We then generated a set of 10 odors, each targeting 8 glomeruli, with the identity of the glomeruli selected to produce sufficient odor diversity (mean glomerular overlap = 4.3, SD = 2.18). We simulated the network receiving each of the 10 odors and generated a spike train for each. We then calculated the correlation between odor pairs in the same manner as for our decorrelation experiments: first, we measured the Pearson correlation between MC firing rates over sliding time windows of length 5 ms (with 50% overlap between windows) at corresponding points in each odor’s spike train. Then, we averaged these correlation values over the time windows comprising the second sniff and took this value to be the overall correlation for an odor pair. After this, we performed a round of neurogenesis, adding in new GCs and determining the connections of the new GCs via the methodology described previously: for each new GC, the probability of connection with each overlapping MC was calculated and sampled to establish the presence or absence of a synapse. We then re-ran our odor simulations with the new network, recording the new odor pair correlations and then performing another round of neurogenesis. Altogether, we repeated this process 10 times (i.e. performed 10 rounds of neurogenesis) and examined how the correlation between odor pairs evolved with each version of the model, as well as the net change in correlation following all rounds of neurogenesis.

We considered 4 separate “modes” of neurogenesis. The first mode, which we labeled the “control” mode, utilized a network consisting of 916 MCs and 13,470 GCs (i.e. with a full GC:MC ratio). In this mode, for each round of neurogenesis, 25% of the GCs in the system were randomly selected to be replaced by newborn GCs, which also were localized randomly in the OB space. In the second mode, which we labeled the “baseline” mode, we modeled a simple form of activity-based selection. We employed the same network as in the control mode, but instead of replacing a random selection of pre-existing GCs, newborn GCs replaced the least active GCs. To do this, once all 10 simulations in a round had been completed, we summed the recorded firing rates of the GCs into a single vector, with each entry representing the aggregated firing of a particular GC. Then, we removed the 25% of GCs with the lowest overall firing rates and replaced them with new GCs, which again were localized randomly in the OB space. In the third mode, which we labeled the “addition” mode, we added new GCs permanently into the system with each round of neurogenesis. Of note, the OB does not appear to increase in cell density indefinitely with neurogenesis, but rather grows throughout life to accommodate new cells [62]. While we did not model this spatial growth directly for the sake of simplicity, we did begin with a smaller network consisting of 864 MCs and 4,320 GCs (1/3 the normal GC:MC ratio) to act as a base structure on which to add new GCs. Thus, when new GCs were added to the system, the pre-existing GCs were retained. In this mode, for each round of neurogenesis we added 864 GCs (1/10 of the remaining number of GCs needed to achieve a full 15:1 ratio of GCs to MCs) and placed them randomly in the OB space. Finally, the fourth “guided addition” mode was conducted similarly to the addition mode, except the newly added GCs were localized near active MCs. To achieve this, following each simulation, we calculated the summed total firing rate across all odors for each MC and divided by the sum of these total firing rates for all MCs to create a probability distribution. For each new GC, we sampled from this distribution to select an MC. Then, the x and y positions of the GC were chosen such that the center of the GC was within 1/10 of the MC’s arbor radius away from the MC’s center, while the z-position of the GC was determined to ensure overlap between the MC and GC. Overall, for each mode we conducted 10 trials of the experiment, with each trial consisting of 10 rounds of neurogenesis.

**Fig 7:**
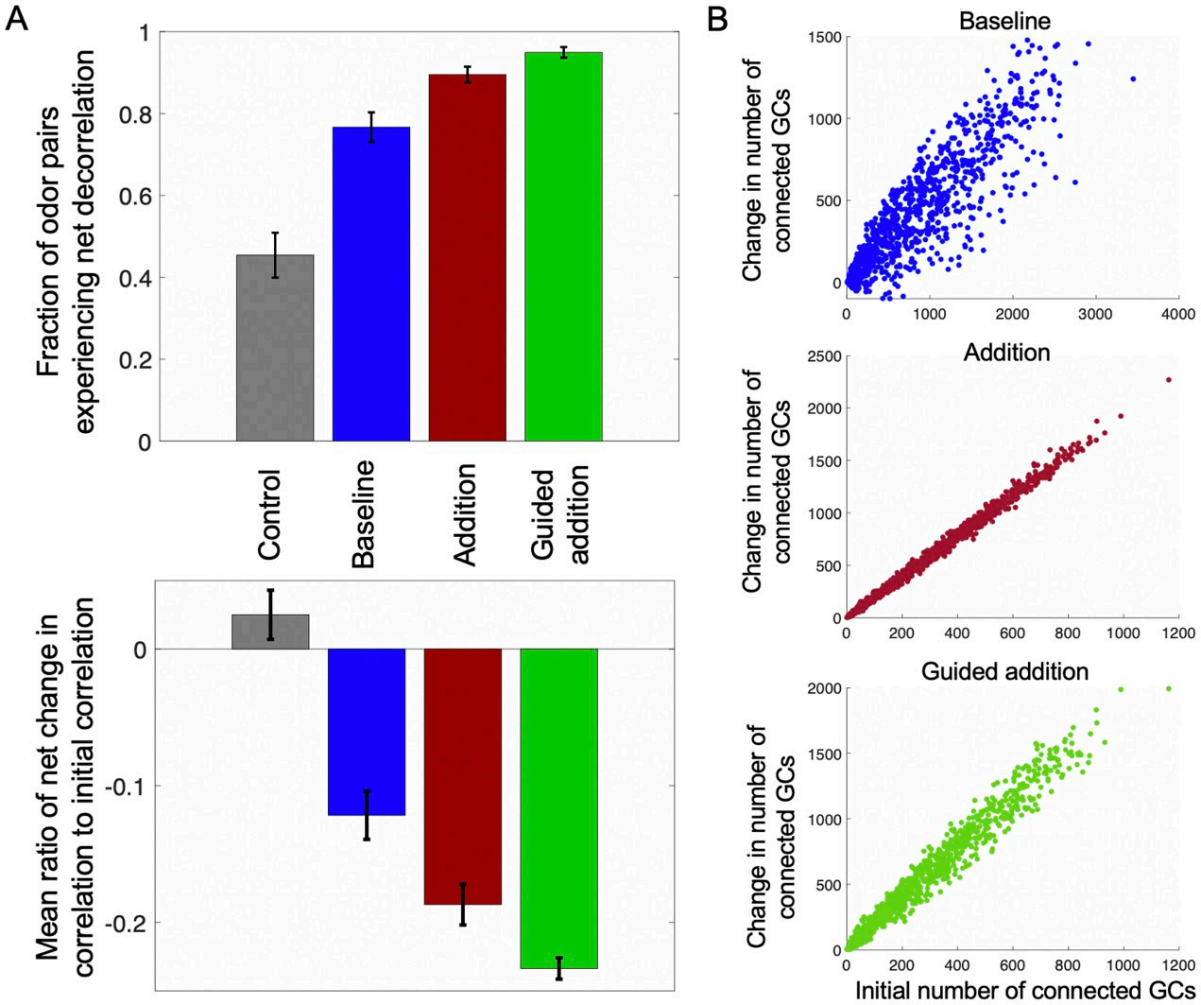
The effects of different modes of neurogenesis on OB structure and function. (A) Different modes of neurogenesis produce different levels of reduction in correlation between odor pairs. On average, the baseline mode (dark blue) produced a 12.16% relative reduction in odor pair correlation (SEM = 0.0177, bottom panel), and 76.7% of odor pairs experienced a net decorrelation (SEM = 0.0361, top panel). The addition mode (dark red) produced an average relative reduction of 18.7% (SEM = 0.0150, bottom panel), and 89.5% of odor pairs were decorrelated at the end of all rounds of neurogenesis (SEM = 0.0193, top panel). The guided addition mode (green) had the strongest effect, leading to an average relative reduction of 23.4% (SEM = 0.0078, bottom panel), with 94.9% of all odor pairs having a net decrease in correlation (SEM = 0.0130). Meanwhile, the control mode (gray) produced little change in the relative reduction (mean = 2.5%, SEM = 0.0180), with 45.4% of odor pairs experiencing net decorrelation (SEM = 0.0548), about what one would expect from chance. Bars indicate the standard error of the mean (SEM). Each mode’s means were averaged from the means of N = 10 trials. (B) Use of a localization rule reduces anatomic constraints on MC connectivity with new GCs. Each panel represents the relationship between a MC’s initial number of connected GCs with the ultimate change in the number of connected GCs following all rounds of neurogenesis.

We considered only odor pairs with a significant initial correlation (>0.1) and examined, following all rounds of neurogenesis, 1) the average change in correlation for each odor pair relative to their initial correlation, and 2) the fraction of odor pairs which experienced a net decorrelation. Unsurprisingly, the control condition, which essentially amounted to the random shuffling of GCs, produced little change in odor correlation; moreover, around 50% of odor pairs experienced a net decrease in correlation, as one would expect from chance (Fig 7A, gray bars). The baseline condition, utilizing activity-dependent selection, also produced results that we expected given previous computational studies of neurogenesis, with odor pairs on average experiencing a relative reduction in correlation, and around 75% of odor pairs experiencing a net decorrelation following all rounds of neurogenesis (Fig 7A, blue bars). Interestingly, however, the addition mode produced an even greater effect than the baseline mode, with odor pairs on average experiencing an even greater mean relative reduction in correlation than the baseline mode, and almost 90% of odor pairs experiencing a net decrease in correlation (Fig 7A, red bars). Finally, the guided addition mode, incorporating a guidance criterion, enhanced the addition mode even further, leading to net decrease in correlation for almost 95% of odor pairs, as well as the highest average relative reduction in correlation among the four modes tested (Fig 7A, green bars). For the latter 3 noncontrol modes, despite odor pairs tending to become less correlated with neurogenesis, there was high variance in odor pair correlation with the evolution of the network; that is, the trend in correlation across the rounds of neurogenesis for most odor pairs was rarely monotonically decreasing (S4 Fig). Overall, we observed that the addition of GCs in and of itself was sufficient to increase the decorrelatory power of the network, but that this power could be further improved by adding an activity-dependent-like localization rule.

Given these results, we were also curious how these different modes of neurogenesis affected the connectivity of the network. We compared the number of each MC’s initial complement of connected GCs with its overall increase or decrease in the number of connected GCs after all rounds of neurogenesis. We found that for all modes other than the control, the more GCs the MC initially connected to, the more subsequent GCs it went on to connect with during succeeding rounds of neurogenesis (examples from single trials in Fig 7B). This suggests that the ability of the network to reshape its connectivity from an anatomical perspective is strongly limited by the pre-existing MC architecture. Even under this constraint, it appears the mere addition of GCs is to a large degree sufficient to decrease the correlation between odors, unsurprising given the GC’s natural tendency to decorrelate (Fig 5C and 5D). However, the use of a localization rule also appears to improve decorrelation by allowing the network to diverge somewhat from these anatomical constraints and to thus adjust the network connectivity to the given odor environment (Fig 7B, top and bottom panels). Thus, even without the ability to change the overall number of GCs, the baseline mode was still able to increase decorrelation between odors, while the guided addition mode was able to draw on both of these sources of decorrelation for the strongest effect.

## Discussion

The functions of the olfactory bulb, which reshape odor representations before they are sent to cortex, emerge from the complex dynamics of a structured network of granule cells, mitral cells and other cell types. Computational models are necessary for determining these collective dynamics, but are challenging to manipulate because of the size and complexity of the network. Many models navigate this challenge by building networks with a reduced number of neurons or GC:MC ratio [10, 11, 13, 14, 19, 22, 23] and either random connectivity or simple distance-dependent functions to generate connectivity [10–22]. These works have replicated several experimentally observed phenomena, such as LFP oscillations [11, 13–15, 17, 20, 23], odor decorrelation [21, 26, 88] and generalization [98], and even odor discrimination in complicated environments [12]; moreover, they have offered potential mechanisms for the relationship between cortex and OB via processes such as feedback and neurogenesis [12, 25, 26, 28, 47, 98]. However, the functional consequences of the anatomical constraints imposed by the OB’s cellular morphology remain unclear. Thus, we developed a modeling approach that employed geometry and dynamical systems theory to integrate realistic details of single cell anatomy [2, 8, 9, 40, 41, 66] and physiology [8, 9, 67, 76], along with empirical information about synaptic architecture [64], into a tractable, yet grounded, computational model of the OB network that simulates the activity of tens of thousands of cells on a standard laptop (parallelization could be further employed for greater speed).

We used this model to replicate previously known experimental and computational results, and also demonstrated a number of new findings that elucidate the role that anatomy plays in OB function: 1) the connectivity between MCs via their shared GCs is distance-dependent in an anatomically constrained manner, and produces inhibitory effects that differ from those seen in standard random network models; 2) anatomy fundamentally shapes the effect of cortical feedback to the OB; and 3) anatomy limits the connectivity profiles of newborn GCs, but despite this, simple incorporation of newborn GCs produces palpable decorrelatory effects without activity-dependent selection. We discuss these points further in the following paragraphs.

### Distance-dependent versus independent GC-mediated inhibition

One study attending to anatomy in large-scale OB modeling is [109], whose authors simulated individual lateral dendrites for each MC along a curved model of the OB, a level of detail limiting the number of MCs to an order of magnitude fewer than in our model. Their connectivity distributions for MCs to GCs and vice versa were Gaussian, while ours are heavily skewed, with a low peak and a long tail (Fig 3). We considered that this difference may arise because MCs residing near the edges of our flat OB have curtailed lateral dendritic fields and hence presumably form fewer connections with GCs, while the curved OB of [109] has a smaller edge for a given surface area, leading to less skew. However, we found that periodic boundary conditions, which lead to *no* curtailed dendritic fields, are still far from having Gaussian connectivity (S3 Fig). The key difference is likely that [109] assumed fixed MC-GC synapse density per length along the MC lateral dendrites. The normal distribution of the MC dendritic field in [109] then implies normally distributed connectivity. Our model includes an additional spatial constraint: MCs “compete” for GCs since GCs each have a limited number of spines. Thus MCs with the most favorable lateral dendritic distributions for a given GC configuration form the most synapses, leading to the exponential distribution for MC connectivity. Indeed, if we counter-factually assumed a fixed distance-independent probability of MC-GC synapses, our model also produced normal connectivity distributions (S2 Fig).

Functionally, these differences manifest in the effects of lateral inhibition. Our results show that, statistically, lateral inhibition is more likely to be stronger over shorter distances as a consequence of the shape of the network’s distance-dependent connectivity. In contrast, [109] specifically demonstrates an example where MCs which are far apart have a significant influence on each other’s firing. The appearance of such long-range inhibition is unlikely in our anatomically grounded model which uses a lower GC:MC ratio of 15:1 (unlike ratios between 20:1 and 100:1 in [109]), and does not assume fully connected dendrites as in [109]. It is conceivable that the prior synaptic tuning performed in [109] to simulate odor learning may allow the sparser inhibitory connections in our model to have a stronger long-range effect. Since we perform no such tuning and presume all synaptic strengths to be of roughly equal magnitude, the network’s natural, distance-dependent architecture determines lateral inhibition. Thus, network remodeling, whether through synaptic plasticity [27] or neurogenesis [36] may be key to overriding the anatomical constraints which would otherwise disfavor long-range interactions. This explanation may also contribute to resolving conflicting reports of the extent of lateral inhibition in the mammalian OB, with some experimental studies reporting that interactions are at least partially short-range [110–113] consistent with our findings, and others reporting distance-independent inhibition [114–116].

### Network influence on global processes

Our model predicts that with a realistic OB network topology and single cell response physiology, gamma oscillations, classically associated with feedback-independent OB activity after odor input [55], only appear with a lower active GC:MC cell ratio than the anatomical proportion. At the full ratio, we see beta oscillations, more commonly associated with activation of cortical feedback to the bulb during odor input [55], an effect that we did not explicitly model. Our results are consistent with previous experiments and models [14, 23, 39] which suggest that gamma oscillations appear when GC activity is reduced due to a lower baseline excitability than suggested by single cell neurophysiology, perhaps due to the influence of centrifugal [117] or deep short-axon cell inhibition [118]. Alternatively, the absence of gamma at high active GC:MC ratio could reflect the possibility that GC activation generally produces oscillations predominantly in the beta range, with other types of EPL interneurons being responsible for gamma oscillations [1]. Additionally, GCs have local and spike-independent processes [6] which may play a role in gamma oscillations [14, 20]; our model utilizes point neurons in order to facilitate large-scale simulation and does not fully capture such processes. Future experimental and theoretical work can separate these possibilities.

The model also suggests that cortical feedback to the bulb will be heavily guided by the existing network structure. Specifically, we found that the MCs affected by feedback to GCs were largely determined by the network configuration, rather than by the GCs targeted. Likewise, the local connectivity to GCs determined which MCs responded most to direct cortical excitation. Similarly, we found that sister MCs originating in the same glomerulus exhibited highly non-overlapping connectivity patterns (Fig 1C). This prediction is consistent with previous experimental studies [3, 4], but differs from many models, which, for simplicity, treat all MCs associated to a glomerulus as equivalent [15, 18, 21, 89, 119–122]. Thus, our results suggest the importance of accurately including network structure for determining the function of individual cells in the OB and their collective behavior, as well as the power of our model in providing a way to probe this relationship.

### Exploring possible mechanisms of neurogenesis

Adult neurogenesis of GCs is critical to OB function, improving odor decorrelation [35, 101, 102] and facilitating olfactory learning and odor memory formation [35, 58–61]. Our longstanding conception of how this process occurs, namely via activity-dependent survival of newborn GCs, which in turn replace pre-existing GCs, has recently been disputed [62]. Specifically, new findings contradict the notion of mass cell death motivating a highly malleable network structure, instead suggesting that GC incorporation into the OB is, for all intents and purposes, permanent. Interestingly, our results demonstrated that the absence of cell death, far from impeding the efficacy of neurogenesis for decorrelating odors, rather promoted it, with the caveat that we did not directly model the physical growth of the OB. Nonetheless, it would appear that the addition of new GCs in and of itself should be sufficient to boost discriminatory power. Intriguingly, however, the inclusion of a simple activity-based localization rule further enhanced the decorrelating effect provided by new GCs. Consequently, it would appear that there exist at least two different avenues by which neurogenesis can facilitate decorrelation—either via remodeling of the OB network, or via growth of its native decorrelating ability via simple addition of GCs.

Although the simplicity of this activity-based localization rule likely limited the degree to which it was able to enhance decorrelation, it did provide clues to what a more effective localization rule might require. Unlike in previous models, which assumed distance-independent connectivity [12, 21, 26], we observed GC localization in our framework to be heavily constrained by the existing network structure, with newborn GCs generally preferring to synapse with MCs which had “advantageous” dendritic distributions that already connected to many GCs (Fig 7B). The use of an activity-based localization criterion, however, tended to reduce this tendency, with the increased spread of values in the top and bottom panels of Fig 7B indicating that at least some GCs were localizing around active MCs, and not necessarily anatomically favorable ones. Thus, the effectiveness of a given localization criterion might be measured by the degree to which it allows newborn GCs to overcome existing anatomic constraints when localizing, thereby preserving flexibility of the OB network in different odor environments. Testing more sophisticated rules, for example optimizing newborn GC placement among pairs or triplets of active MCs, might provide the next steps in maximizing this flexibility and, in turn, the effectiveness of neurogenesis from a theoretical perspective.

### Extending the model

Future expansion of the model will necessitate incorporating the plasticity of cell-cell interactions as well as refining internal cell dynamics. In particular, the OB is known to undergo constant remodeling throughout its lifetime due to the effects of synaptic plasticity [27, 123]. Moreover, overall bulbar activity is dependent on the internal dynamics and excitability of both MCs and GCs. For example, subthreshold oscillations directly influence synchronicity between MCs, which in turn affects odor perception [13, 124–126]; thus physiologic fluctuations in the frequency of these oscillations could have palpable effects on OB function, which more advanced modeling of MCs could help realize (e.g. [43]). Such factors are also relevant to neurogenesis, as adult newborn GCs demonstrate increased excitability and enhanced synaptic plasticity, which directly impact olfactory learning and likely have a strong effect on how and where these cells incorporate into the OB network [29, 31, 101, 127, 128]. Altogether, inter- and intracellular processes play a critical role in the adaptation of the OB to a given odor environment. Consequently, their incorporation will be a critical next step in the evolution of this model and its utility in further unraveling the fundamental relationship between structure and function.

It will be further interesting to extend our model by introducing other OB cell types, notably tufted cells (TCs) and their corresponding superficial granule cells. TCs form the second major population of excitatory neurons in the OB and have different anatomical and electrophysiological properties from MCs [2, 9, 40, 41]. Moreover, they may have significantly different functionality from MCs in odor processing [93, 129–131]. For their part, superficial GCs also have anatomical and electrophysiological properties which separate them from deep GCs [8, 40, 66]. Indeed, based upon their location in the EPL, they likely form connections with type II MCs in addition to TCs, and, in our model, their absence may potentially explain the relatively lower number of GCs connected to type II MCs as compared to type I MCs. Thus, the addition of such cell types to our model will facilitate exploration of the differences between MCs and TCs as well as between the two MC types. Perhaps such studies will shed light on a classic question: why do neural circuits like the OB need so many different cell types with different properties to carry out their functions, rather than simply having more complex circuitry connecting fewer functional types [132]?

## Methods

All values were derived, where possible, from measurements of the murine olfactory bulb [4, 8, 9, 33, 133], the chief exception being the derivation of MC lateral dendritic density, which was based on images from study of rabbit [40] and rat [41].

### Mitral cell lateral dendritic density

Analyzing *camera lucida* drawings of mitral cells from [40] and [41], we fit a function of the following form for the total dendritic length contained within a circle of radius *r*:

$$\begin{array}{c} f\left(r\right)=\alpha \;{\text{tan}}^{-1}\left(kr+\beta \right)+C \end{array}$$ (22)

Then by default:

$$\begin{array}{c} f\left(0\right)=\alpha \;{\text{tan}}^{-1}\left(\beta \right)+C=0 \end{array}$$

and:

$$\begin{array}{c} \beta =\text{tan}(-C/\alpha ) \end{array}$$

So Eq 22 becomes:

$$\begin{array}{c} f\left(r\right)=\alpha \;{\text{tan}}^{-1}\left(kr-\text{tan}\left(\frac{C}{\alpha }\right)\right)+C \end{array}$$ (23)

where *r* ∈ [0, *r*_max_] and $\frac{C}{\alpha }\in \left[0,\frac{\pi }{2}\right)$. Replacing for convenience *C* with *mα*, Eq 23 then becomes:

$$\begin{array}{c} f\left(r\right)=\alpha \;{\text{tan}}^{-1}\left(kr-\text{tan}\left(m\right)\right)+m\alpha \end{array}$$ (24)

with $m\in [0,\frac{\pi }{2})$

Now for an annulus of thickness *ϵ*, the ratio of the additional length of dendrite encapsulated in the annulus to the area of the whole annulus is:

$$\begin{array}{c} \rho_{m}\left(r\right)=\frac{f(r+\epsilon )-f(r)}{\epsilon (2\pi r+\pi \epsilon )} \end{array}$$

In the limit as *ϵ* goes to zero, we see that this equation simply becomes:

$$\begin{array}{c} \rho_{m}\left(r\right)=\frac{1}{2\pi r}\frac{df}{dr} \end{array}$$

Plugging in *f*(*r*), Eq 24 for the dendritic density finally becomes Eq 1:

$$\begin{array}{c} \rho_{m}\left(r\right)=\frac{\alpha k/2\pi r}{1+{\left(kr-\text{tan}\left(m\right)\right)}^{2}} \end{array}$$ (25)

*α*, *k*, and *m* are defined in terms of the variables *γ*, *ξ*, and the maximum radius *r*_max_. γ ∽ Uniform(0.2, 0.3) describes the fraction of *r*_max_ where the maximum of $\frac{df}{dr}$ occurs. ξ ∽ Uniform(1/3, 4/5) represents the fraction of the maximum value of $\frac{df}{dr}$ at *r* = 0. Additionally, we assume that the total length of dendrite *L* for a given MC is proportional to the area of that MC’s dendritic field, such that:

$$\begin{array}{c} L=w\pi r_{max}^{2} \end{array}$$ (26)

where *w* has units of *μ*m^−1^ and ∽ Uniform(0.00255, 0.00510) for each MC. Then, since the maximum value of $\frac{df}{dr}$ is *αk* and occurs at *r* = tan(*m*)/*k*, it follows that:

$$\begin{array}{c} \eta =\frac{\text{tan}(m)}{kr_{\text{max}}} \end{array}$$ (27)

$$\begin{array}{c} \xi =\frac{1}{1+\text{tan}{\left(m\right)}^{2}} \end{array}$$ (28)

and the values of *m*, *k*, and *α* can be re-expressed as:

$$\begin{array}{c} m={\text{tan}}^{-1}\left(\sqrt{\frac{1}{\xi }-1}\right) \end{array}$$ (29)

$$\begin{array}{c} k=\frac{\text{tan}(m)}{\eta r_{\text{max}}} \end{array}$$ (30)

$$\begin{array}{c} \alpha =\frac{L}{{\text{tan}}^{-1}\left(kr_{\text{max}}-\text{tan}\left(m\right)\right)+m} \end{array}$$ (31)

### Granule cell spine density

The equation of spine density (in spines per unit volume) as defined in Eq 2 was:

$$\begin{array}{c} \rho_{g}\left(z\right)=\frac{N_{s}\left(z\right)}{\pi r{\left(z\right)}^{2}} \end{array}$$ (32)

For *z* ∈ (*z*_0_, *z*_max_], where *z*_max_ and *z*_0_ are the maximum height and bottom of the dendritic tree respectively. *r*(*z*), the radius as a function of height, was simply determined using similar triangles:

$$\begin{array}{c} r\left(z\right)=\frac{r_{\text{max}}}{z_{\text{max}}-z_{0}}\left(z-z_{0}\right) \end{array}$$ (33)

*N*_*s*_(*z*), which describes the linear spine density as a function of height, was assumed a parabola as an approximation of the linear spine densities found in [8] and [40]:

$$\begin{array}{c} N_{s}\left(z\right)=-a\left(z-z_{0}\right)\left(z-z_{\text{max}}\right) \end{array}$$ (34)

Where *a* is a constant to be determined as follows. Since *N*_*s*_(*z*) is subject to the constraint:

$$\begin{array}{c} \int_{z_{0}}^{z_{\text{max}}}N_{s}\left(z\right)dz=S \end{array}$$

where *S* is the total number of spines on the cone, then:

$$\begin{array}{c} a=\frac{6S}{{\left(z_{\text{max}}-z_{0}\right)}^{3}} \end{array}$$ (35)

And Eq 34 thus becomes:

$$\begin{array}{c} N_{s}\left(z\right)=\frac{-6S}{{\left(z_{\text{max}}-z_{0}\right)}^{3}}\left(z-z_{0}\right)\left(z-z_{\text{max}}\right) \end{array}$$ (36)

Ultimately, substituting in Eq 33 for *r*(*z*), Eq 36 finally becomes 3:

$$\begin{array}{c} \rho_{g}\left(z\right)=\frac{-6S}{\pi r_{\text{max}}^{2}\left(z_{\text{max}}-z_{0}\right)}(\frac{z-z_{\text{max}}}{z-z_{0}}) \end{array}$$ (37)

### Calculating the overlap dendritic length

From Eq 25, *L*, the total length of dendrite contained in the overlap between an MC and GC, is:

$$\begin{array}{c} L=\int_{A(z_{m})}\rho_{m}\left(r\right)dA=\underset{A(z_{m})}{\;\int \int \;}\frac{\alpha k/2\pi r}{1+{\left(kr-\text{tan}\left(m\right)\right)}^{2}}rdrd\theta \end{array}$$ (38)

Calculating this integral is dependent on *s*, the distance between the center of the MC field and the GC field, as well as on *r*_*m*_, the radius of the MC, and *r*_*g*_, the radius of the GC at *z*_*m*_, the MC height and thus the height of the intersection. Below we detail how the integral changes as the two fields are drawn closer together.

For all cases, if *s* ≥ *r*_*g*_ + *r*_*m*_, or if *z*_*m*_ is out of range of the GC cone (*i.e.* the MC and GC do not overlap), then by default:

$$\begin{array}{c} L=0 \end{array}$$ (39)

Again for all cases, if $\sqrt{r_{g}^{2}+r_{m}^{2}}\leq s<\left(r_{g}+r_{m}\right)$, then

$$\begin{array}{c} L=2\int_{0}^{\mu }\int_{g(\theta )}^{r_{m}}\rho_{m}\left(r\right)rdrd\theta \end{array}$$ (40)

where:

$$\begin{array}{c} \mu ={\text{cos}}^{-1}(\frac{r_{m}^{2}+s^{2}-r_{g}^{2}}{2r_{m}s}) \end{array}$$ (41)

$$\begin{array}{c} g\left(\theta \right)=s\;\text{cos}\left(\theta \right)-\sqrt{r_{g}^{2}-s^{2}{\text{sin}}^{2}\left(\theta \right)} \end{array}$$ (42)

the latter of which is just the equation in polar coordinates for a circle a distance *s* from the origin. Since the problem is symmetric, we multiplied the integral by 2 and integrated from 0 rather than integrate from −*μ* to *μ*. Note that we adopted this strategy for all integrals except the case where the GC field overlapped the center of the MC field, in which case we integrated over all *θ* (see below).

Moving forward, the integrals depended on the relative sizes of *r*_*g*_ and *r*_*m*_, so below we consider the relevant cases separately. Before continuing, we must define two further quantities:

$$\begin{array}{c} \gamma ={\text{sin}}^{-1}\left(r_{g}/s\right) \end{array}$$ (43)

$$\begin{array}{c} g^{\prime }\left(\theta \right)=s\;\text{cos}\left(\theta \right)+\sqrt{r_{g}^{2}-s^{2}{\text{sin}}^{2}\left(\theta \right)} \end{array}$$ (44)

where the latter is again the equation in polar coordinates for a circle a distance *s* from the origin, but integrated in the opposite direction from *g*(*θ*).

**Case 1**: ***r*_*m*_ > 2*r*_*g*_**

If $r_{m}-r_{g}\leq s<\sqrt{r_{g}^{2}+r_{m}^{2}}$

$$\begin{array}{c} L=2(\int_{0}^{\mu }\int_{g(\theta )}^{r_{m}}\rho_{m}\left(r\right)rdrd\theta +\int_{\mu }^{\gamma }\int_{g(\theta )}^{g^{\prime }\left(\theta \right)}\rho_{m}\left(r\right)rdrd\theta ) \end{array}$$ (45)

If *r*_*g*_ ≤ *s* < *r*_*m*_ − *r*_*g*_

$$\begin{array}{c} L=2\int_{0}^{\gamma }\int_{g(\theta )}^{g^{\prime }\left(\theta \right)}\rho_{m}\left(r\right)rdrd\theta \end{array}$$ (46)

If 0 ≤ *s* < *r*_*g*_

$$\begin{array}{c} L=\int_{0}^{2\pi }\int_{0}^{g^{\prime }\left(\theta \right)}\rho_{m}\left(r\right)rdrd\theta \end{array}$$ (47)

**Case 2**: **2*r*_*g*_ > *r*_*m*_ > *r*_*g*_**

If $r_{g}\leq s<\sqrt{r_{g}^{2}+r_{m}^{2}}$

$$\begin{array}{c} L=2(\int_{0}^{\mu }\int_{g(\theta )}^{r_{m}}\rho_{m}\left(r\right)rdrd\theta +\int_{\mu }^{\gamma }\int_{g(\theta )}^{g^{\prime }\left(\theta \right)}\rho_{m}\left(r\right)rdrd\theta ) \end{array}$$ (48)

If *r*_*m*_ − *r*_*g*_ ≤ *s* < *r*_*g*_

$$\begin{array}{c} L=2(\int_{\mu }^{\pi }\int_{0}^{g^{\prime }\left(\theta \right)}\rho_{m}\left(r\right)rdrd\theta +\int_{0}^{\mu }\int_{0}^{r_{m}}\rho_{m}\left(r\right)rdrd\theta ) \end{array}$$ (49)

If 0 ≤ *s* < *r*_*m*_ − *r*_*g*_

$$\begin{array}{c} L=\int_{0}^{2\pi }\int_{0}^{g^{\prime }\left(\theta \right)}\rho_{m}\left(r\right)rdrd\theta \end{array}$$ (50)

**Case 3**: ***r*_*g*_ > *r*_*m*_**

If $r_{g}\leq s<\sqrt{r_{g}^{2}+r_{m}^{2}}$

$$\begin{array}{c} L=2(\int_{0}^{\mu }\int_{g(\theta )}^{r_{m}}\rho_{m}\left(r\right)rdrd\theta +\int_{\mu }^{\gamma }\int_{g(\theta )}^{g^{\prime }\left(\theta \right)}\rho_{m}\left(r\right)rdrd\theta ) \end{array}$$ (51)

If *r*_*g*_ − *r*_*m*_ ≤ *s* < *r*_*g*_:

$$\begin{array}{c} L=2(\int_{\mu }^{\pi }\int_{0}^{g^{\prime }\left(\theta \right)}\rho_{m}\left(r\right)rdrd\theta +\int_{0}^{\mu }\int_{0}^{r_{m}}\rho_{m}\left(r\right)rdrd\theta ) \end{array}$$ (52)

If 0 ≤ *s* < *r*_*g*_ − *r*_*m*_:

$$\begin{array}{c} L=\int_{0}^{2\pi }\int_{0}^{r_{m}}\rho_{m}\left(r\right)rdrd\theta \end{array}$$ (53)

#### Layers of the OB space

We measured the average thickness of the external plexiform layer (EPL), mitral cell layer (MCL), and internal plexiform layer (IPL) from *camera lucida* images in supplementary material from [8] and [9]:

**Table:** 

**Layer**	**Thickness (in *μ*m)**
EPL (*θ*_EPL_)	131
MCL (*θ*_MCL_)	36
IPL (*θ*_IPL_)	27
Total (*θ*)	194

#### Glomerular density and distribution

For the purpose of calculating the area density of the glomerular projections (henceforth referred to as simply ‘glomeruli’) on the EPL, we assumed the EPL to be a flat (with thus no difference in the surface areas of the top and bottom surfaces), 3-dimensional space with a volume of around 1.5 mm^3^ [134]. We also assumed the thickness of the EPL to be uniform (although in general there is considerable variation in thickness over the bulb), and so by dividing this volume by *θ*_EPL_, we arrived at an area of 1.145 x 10^7^ *μ*m^2^. We assumed 1800 glomeruli per olfactory bulb [133, 135], leading to an area density *ρ*_glom_ of 157 glomeruli/mm^2^. Thus, for the network, $\rho_{\text{glom}}\pi r_{c}^{2}$ glomeruli were placed uniformly randomly in the x-y plane within a radius of *r*_*c*_.

#### Mitral cell distribution

A number of MCs drawn from Uniform(15, 25) was placed around each glomerulus. For a glomerulus located at (*x*_glom_, *y*_glom_), the location of one of these MCs in the x-y plane was (*x*_glom_ + *r* cos *θ*, *y*_glom_ + *r* sin *θ*), where *r* (in *μ*m) ∽ Logistic (*μ* = 78.4, *s* = 23.1) for *r* ∈ [0, 300] [4], and *θ* ∽ Uniform (0, 2*π*). The radius of each MC was drawn from Uniform(75, 800).

MCs were either assigned as type I with $\frac{2}{3}$ probability or type II with $\frac{1}{3}$ probability [2]. The z-positions for each cell type were drawn from the following distributions:

**Table:** 

	**Type I**	**Type II**
Z-position	*θ*_IPL_ + *θ*_MCL_ + $\text{Uniform}(0,\frac{1}{2}\theta_{\text{EPL}})$	*θ*_IPL_ + *θ*_MCL_ + $\text{Uniform}(\frac{2}{5}\theta_{\text{EPL}},\frac{4}{5}\theta_{\text{EPL}})$

#### Granule cell distribution

We specifically modeled deep granule cells, which preferentially interact with mitral cells [8]. GCs were distributed randomly in the xy-plane such that the x and y positions of their bottom vertices were distributed uniformly randomly. The maximum radius, top and bottom z-positions, and xy-distance of the center of the top face from the bottom vertex were drawn from the following distributions (in *μ*m):

**Table:** 

Maximum radius (*r*_max_)	Normal(83, 28) for *r* ∈ [30, 160]
Bottom z-position (vertex)	Uniform(0, *θ*_IPL_ + *θ*_MCL_)
Top z-position (face)	*θ*_IPL_ + *θ*_MCL_ + $\text{Uniform}(\frac{1}{2}\theta_{\text{EPL}},\theta_{\text{EPL}})$
Vertex to face-center projection distance	Uniform(0, 50)

Finally, the number of spines *S* was drawn from a uniform distribution with bounds determined by the volume of the cone. The equation for the bounds was of the form:

$$\begin{array}{c} a\;{\text{tan}}^{-1}\left(bV\right) \end{array}$$ (54)

where *V* is the volume. For the lower bound, *a* = 39.31 and *b* = 1.043 * 10^−5^, while for the upper bound, *a* = 357.7 and *b* = 2.653 * 10^−6^.

### Experimental procedures

#### Skewed normal distribution

A skewed normal distribution has a PDF described by the following equation:

$$\begin{array}{c} f\left(x\right)=\frac{2}{\omega \sqrt{2\pi }}e^{-\frac{{\left(x-\xi \right)}^{2}}{2\omega^{2}}}\int_{-\infty }^{\zeta (\frac{x-\xi }{\omega })}\frac{1}{\sqrt{2\pi }}e^{-\frac{t^{2}}{2}}\;dt \end{array}$$ (55)

where *ω* is the scale parameter, *ζ* is the shape parameter, and *ξ* describes the shift of the distribution.

#### Local field potential oscillations

After the LFP signal was calculated, we passed it through a 6th-order low-pass Butterworth filter with cutoff of 200 Hz and then detrended the signal. We removed the first 200 ms of the signal to roughly isolate its steady-state. We then used Welch’s power spectral density estimate to calculate the power spectrum, utilizing a window size of 400 ms with 50% overlap between windows.

#### Lateral inhibition

MC pairs (here denoted cells A and B) were selected from the network that had a number of connected GCs within 75 of the average for all MCs and whose z-positions lay within 5 *μ*m of each other. During the first simulation, cell A was fed 700 pA of direct current for 1 s (with 100 ms of unrecorded padding time at the beginning of the simulation to allow the cell to activate from rest), and the consequent firing rate for cell A was measured over that 1 s. During the second simulation, cell A was fed 700 pA of direct current while cell B was fed 750 pA of direct current, and the firing rate for cell A was measured again over that 1 s. This process was repeated for 1,436 different pairs of MCs to cover a wide range of inter-cell distances.

#### Decorrelation

For simulation of a given odor, each MC in each glomerulus received input current of the form:

$$\begin{array}{c} I\left(t\right)=\frac{I_{0}}{2}+\frac{I_{0}}{4}\left(\text{sin}\;\left(2\pi ft-\phi \right)+1\right) \end{array}$$ (56)

For the MCs belonging to each glomerulus, *I*_0_ was drawn from $N(I_{\text{mean}},I_{\text{mean}}/5)$ pA. If the glomerulus was one which was designated to receive odor, *I*_mean_ was drawn from a uniform distribution between 400 and 600 pA, while for all other glomeruli, *I*_mean_ was drawn from a uniform distribution between 0 and 150 pA. The phase *ϕ* was drawn as for the LFP experiments: we first drew an initial mean glomerular phase *p*_*g*_ uniformly from 0 to 2*π* for each glomerulus. Then for each MC in a glomerulus we drew *ϕ* from a Gaussian with mean *p*_*g*_ and standard deviation *π*/4.

Six odors were generated which each targeted 30 glomeruli and which had varying degrees of glomerular overlap (between 5 and 25 shared glomeruli, mean = 18), such that the strength and phase of the input for odor-receiving glomeruli were identical; meanwhile, strength and phase for the different glomeruli and for all other non-odor-receiving glomeruli were different. Odor presentation was simulated for 6 sniffs at 6 Hz (i.e. 1 second of in-simulation time) for each odor individually. Then, the spike time series for each odor was divided into windows of time length *T*, with 50% overlap between windows and the firing rate of the MCs for each odor was computed over each interval. Then the Pearson correlation was computed between the firing rates of corresponding intervals for each pair of odors (*n* = 15). The value of *T* was varied to examine how the correlation time course depended on the timescale.

#### Cortical feedback to GCs

In each experiment, an odor was generated which targeted 35 glomeruli. Odor currents were simulated as in the decorrelation experiment, for a total of 2 sniffs (1/3 of a second real-time). We examined only the second sniff (since network dynamics appear to stabilize after one sniff). For baseline, odor input alone was presented, and a sequence of firing rates for each odor-receiving MC was calculated over 10 ms windows, with 50% overlap between successive time windows. For the first condition, excitatory feedback was added to a subset of GCs in the form of 50 pA of constant current, with the subset of GCs being either 0.1%, 1%, 5%, 10%, 15%, or 20% of all GCs (rounded to the nearest whole number). A sequence of firing rates was calculated as for the baseline condition, and the baseline firing rate sequence was subtracted from this new firing rate sequence to generate a sequence of firing rate changes over time. For the second condition, feedback was again presented but to a set of GCs non-overlapping with the first set, and a new sequence of firing rate changes from baseline was generated. We computed the Pearson correlation between the first and second conditions of these firing rate changes for each time window, and then calculated the mean correlation. For the third condition, a new arrangement of GCs was generated around the same MCs and the experiment was repeated, with the correlation of the changes in firing rates being computed between the results of the first condition and this new condition.

#### Cortical feedback to MCs

These experiments were conducted similarly to the previous feedback experiments. During the feedback condition, instead of feedback current to the GCs, constant excitatory feedback current was delivered to 0.2 of all MCs, with the strength of the current for each MC being drawn from Normal(200, 20) pA. Data in the figure was compiled from *n* = 5 trials, each with a different odor and feedback pattern.

#### Neurogenesis

For the simulations, odors were modeled similarly to the decorrelation experiments. For each glomerulus *g*, a strength *I*_*g*_ was drawn from Uniform(150,600) or Uniform (0,150) for odor and non-odor receiving glomeruli, respectively; and a phase *p*_*g*_ was drawn from Uniform(0, 2*π*). Then for each MC in a given glomerulus, a number *I*_0_ and phase *ϕ* were drawn from Normal(*I*_*g*_, *I*_*g*_/5) and Normal(*p*_*g*_, *π*/4) respectively, and the input current was simulated as before:

$$\begin{array}{c} I\left(t\right)=\frac{I_{0}}{2}+\frac{I_{0}}{4}\left(\text{sin}\;\left(2\pi ft-\phi \right)+1\right) \end{array}$$ (57)

Each odor targeted 8 glomeruli, with the identity of the glomeruli selected to produce sufficient odor diversity (mean glomerular overlap = 4.3, SD = 2.18). We set *f* equal to 6 Hz to represent sniffing [136] and simulated each odor in our base system for 0.33 s of in-simulation time, equal to 2 sniffs and sufficient to achieve steady state conditions by the second sniff.

### Simulation

All simulations were done in MATLAB versions R2017, R2018, or R2019 via a forward Euler method with time step = 0.1 ms. Usage of a shorter time step demonstrated little change in the results (S5 Fig).

## Supporting information

S1 FigDistribution of sister MC somata with relation to glomerulus.Distribution of sister MC somata with relation to their parent glomerulus from [4]. This distribution was well fit by a logistic function of the form $1/\left(1+\text{exp}\left(\frac{-(x-m)}{s}\right)\right)+1/\left(1+\text{exp}\left(\frac{m}{s}\right)\right)$, with *m* = 78.4 and *s* = 23.1 (*r*^2^ value = 0.998), and the constant term forcing the function through the origin, since we assume that no MCs are encapsulated by a circle of radius 0.(TIF)

S2 Fig Lateral inhibition and connectivity in the distance-independent network. (A) When the probability of connectivity is independent of distance, the number of shared GCs (A) and strength of lateral inhibition (B) are also both independent of distance. Distributions of connectivity are Gaussian for both (C) MCs and (D) GCs.
(TIF)

S3 Fig Connectivity in the periodic network. Distributions of (A) MC and (B) GC connectivity are right-shifted compared to those of the bounded network.
(TIF)

S4 Fig Evolution of correlation with neurogenesis. Evolution of correlation for 3 odor pairs from one trial of the baseline mode (A), the addition mode (B), and the guided addition mode (C).
(TIF)

S5 Fig Sample set of results repeated with TS = 0.05 ms. Here we demonstrate a sample set of results utilizing a shorter time step of 0.05 ms. The results are generally robust to the shortened time step. (A) The top and bottom panels correspond to the left and right panels of Fig 4, respectively. (B) This panel corresponds to Fig 5B. (C) The top and bottom panels correspond to Fig 5C and 5D, respectively. (D) The top and bottom panels correspond to Fig 6A and 6B, respectively.
(TIF)
