# Supplementary figures and images for: Connectivity and dynamics in the olfactory bulb
Source: PLoS Comput Biol. 2022 Feb 7;18(2):e1009856. doi: 10.1371/journal.pcbi.1009856 (PMC8853646; doi:10.1371/journal.pcbi.1009856)

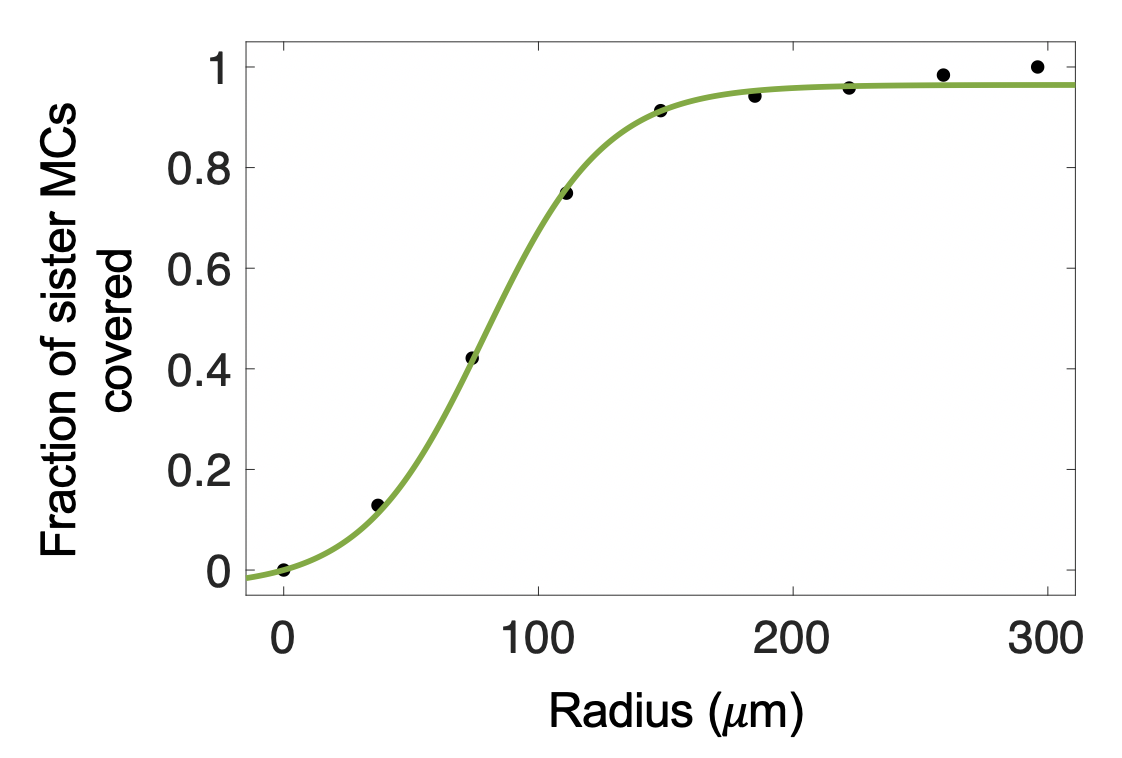

Supplement: S1 Fig — Distribution of sister MC somata with relation to their parent glomerulus from [4]. This distribution was well fit by a logistic function of the form 1/(1+exp(-(x-m)s))+1/(1+exp(ms)), with m = 78.4 and s = 23.1 (r2 value = 0.998), and the constant term forcing the function through the origin, since we assume that no MCs are encapsulated by a circle of radius 0. (TIF) [file pcbi.1009856.s001.tif]

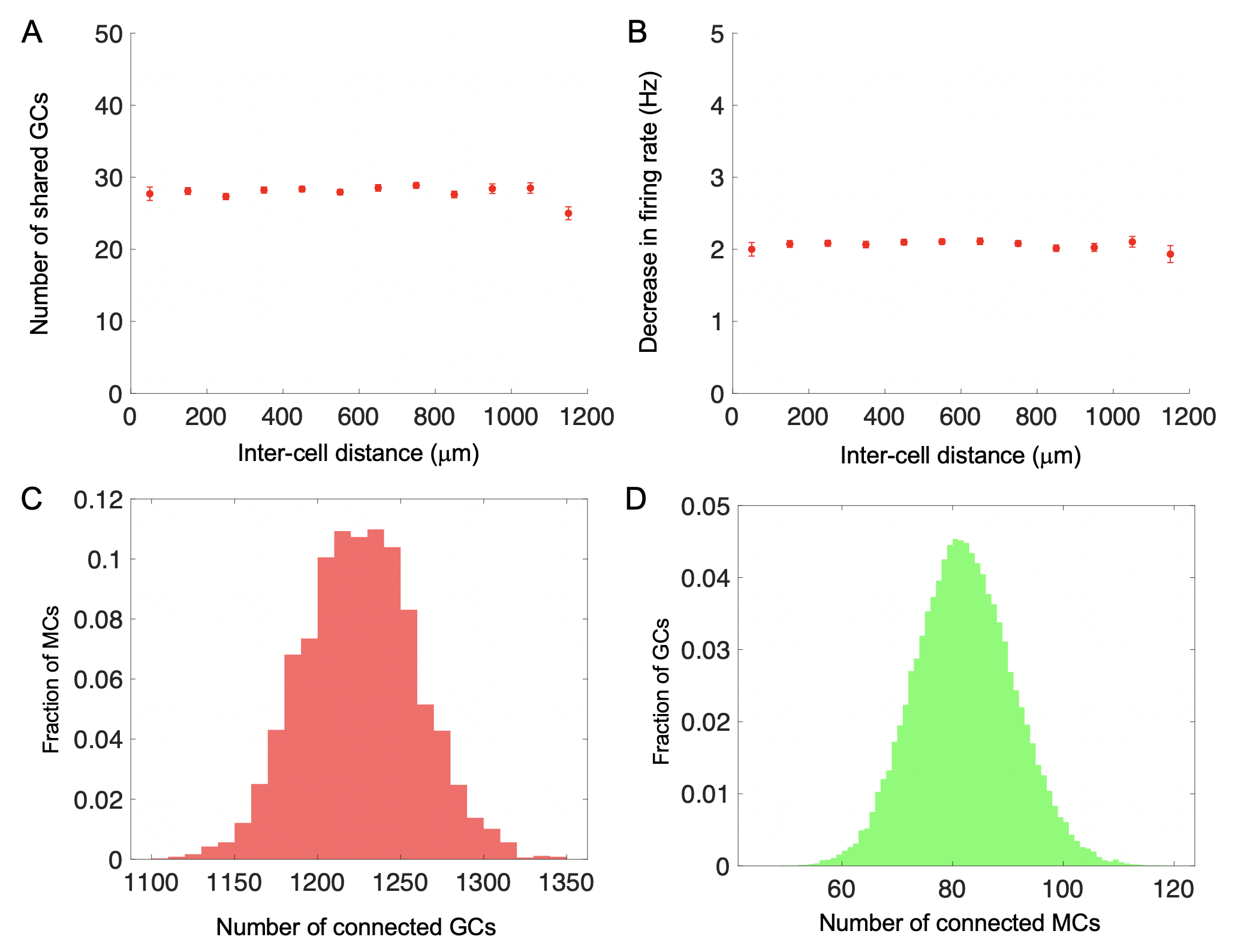

Supplement: S2 Fig — (A) When the probability of connectivity is independent of distance, the number of shared GCs (A) and strength of lateral inhibition (B) are also both independent of distance. Distributions of connectivity are Gaussian for both (C) MCs and (D) GCs. (TIF) [file pcbi.1009856.s002.tif]

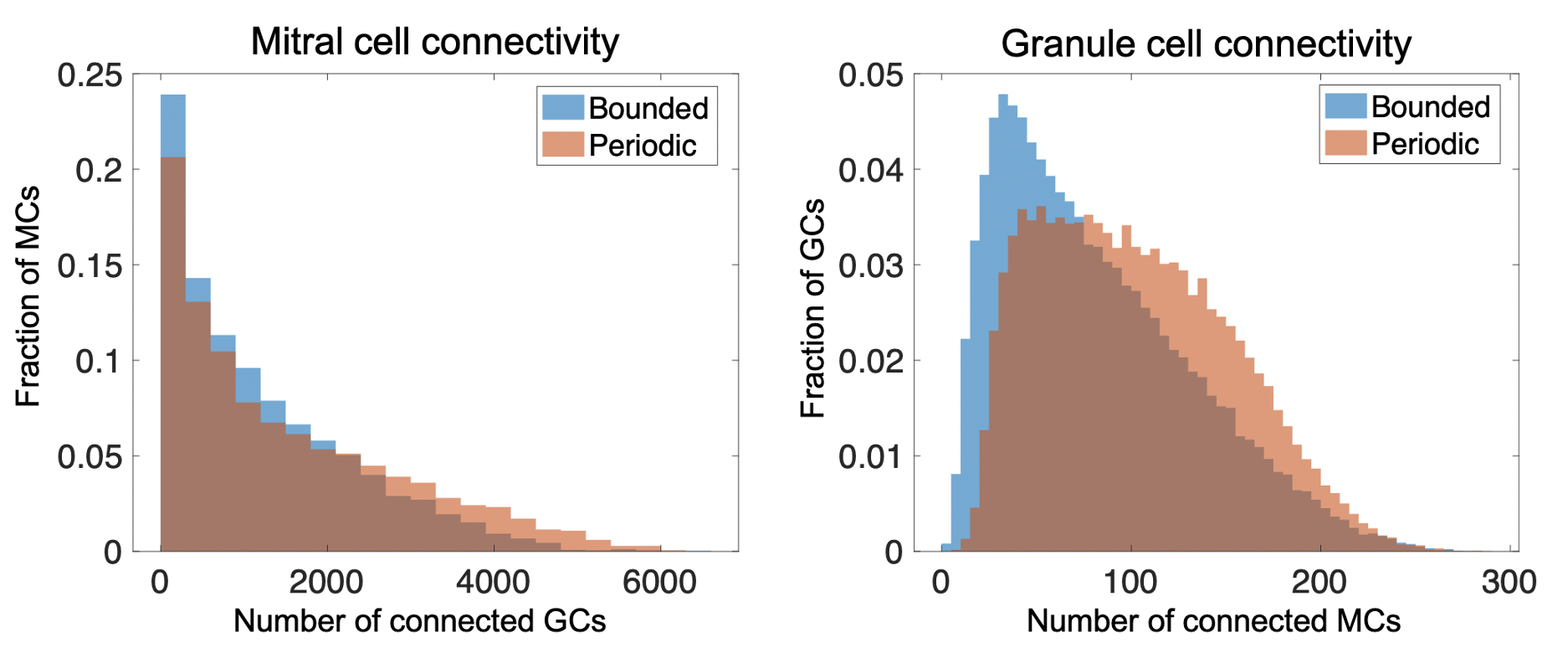

Supplement: S3 Fig — Distributions of (A) MC and (B) GC connectivity are right-shifted compared to those of the bounded network. (TIF) [file pcbi.1009856.s003.tif]

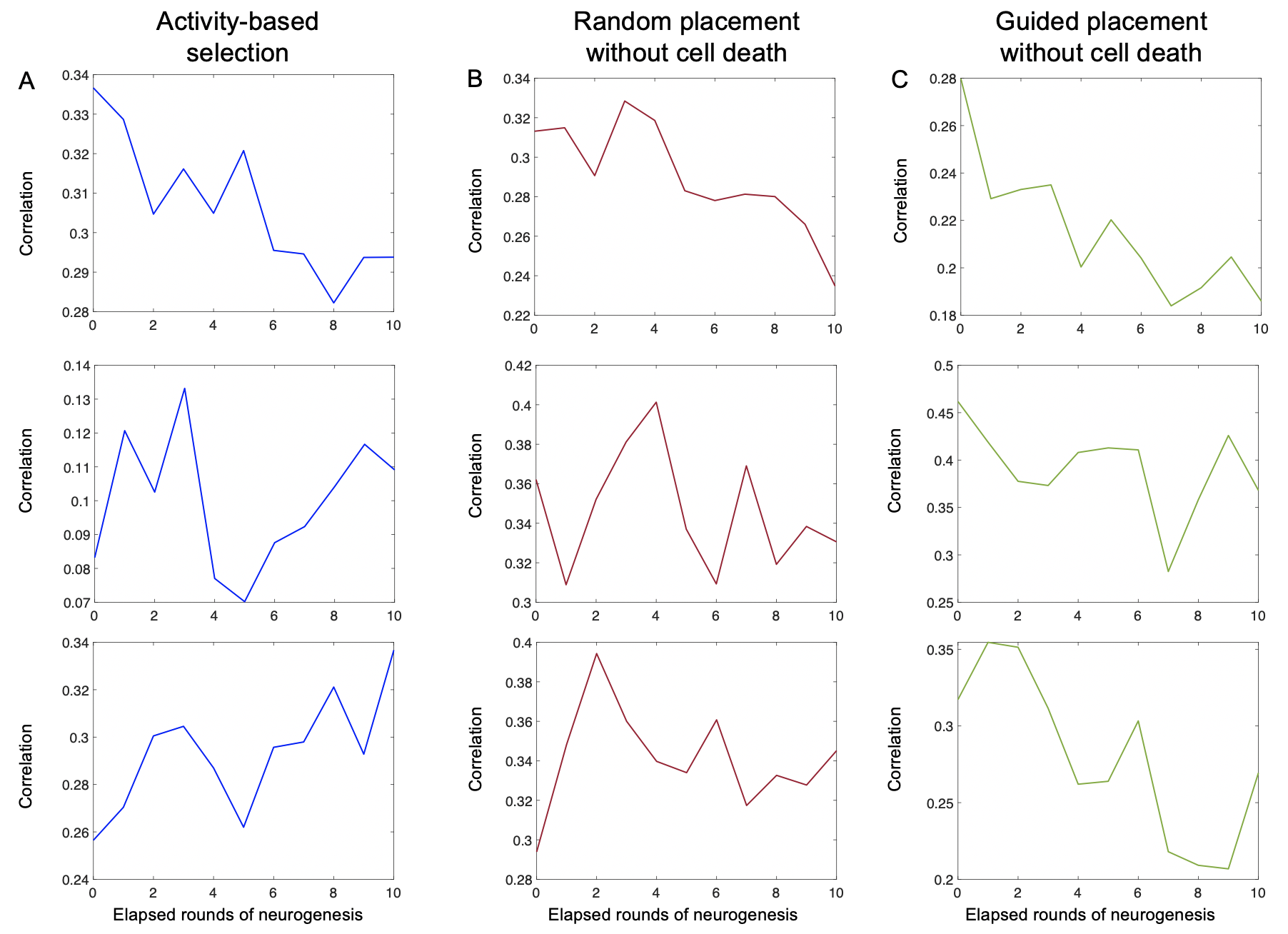

Supplement: S4 Fig — Evolution of correlation for 3 odor pairs from one trial of the baseline mode (A), the addition mode (B), and the guided addition mode (C). (TIF) [file pcbi.1009856.s004.tif]

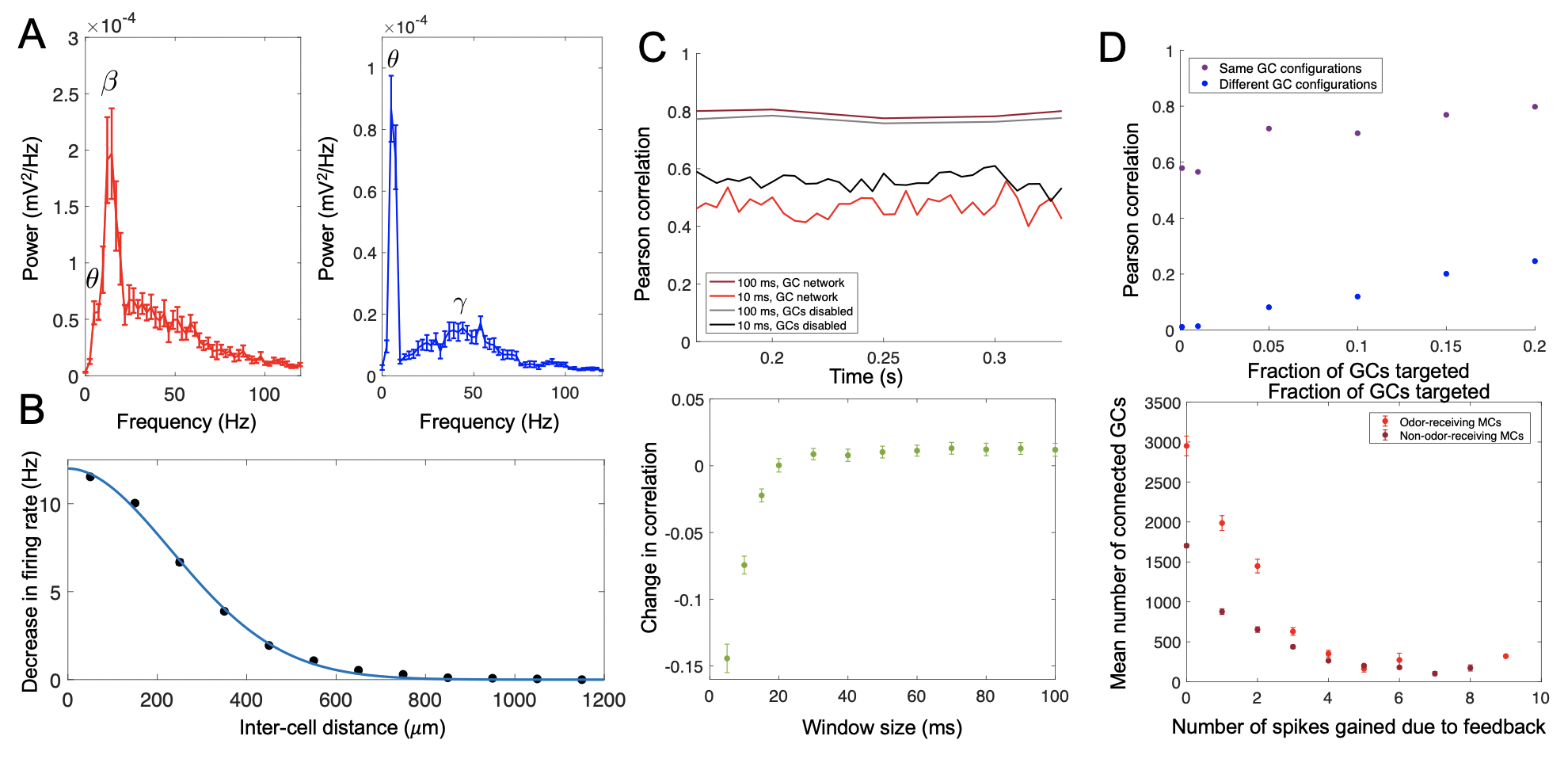

Supplement: S5 Fig — Here we demonstrate a sample set of results utilizing a shorter time step of 0.05 ms. The results are generally robust to the shortened time step. (A) The top and bottom panels correspond to the left and right panels of Fig 4, respectively. (B) This panel corresponds to Fig 5B. (C) The top and bottom panels correspond to Fig 5C and 5D, respectively. (D) The top and bottom panels correspond to Fig 6A and 6B, respectively. (TIF) [file pcbi.1009856.s005.tif]
